# Current Awareness on Comparative and Functional Genomics

**DOI:** 10.1002/cfg.118

**Published:** 2002-06


**Comparative and Functional Genomics**
*Comp. Funct. Genom.* 2002; **3:** 293–304
**Published online in Wiley InterScience (www.interscience.wiley.com). DOI:**10.1002/cfg.118

### Current awareness on comparative and functional genomics

### 1 Reviews & symposia

2001. Special issue on genetics and genomics in molecular medicine. *Trends Mol Med* **7:** (11)
2001. Special issue: Bioinformatics of Genome Regulation and Structure, August 7-11, 2000 - Novosibirsk, Russia. *Mol Biol-Engl Tr* **35:** (6)
2001. Special issue: Genomes and evolution. *Curr Opin Genet Dev* **11:** (6)
2001. Special issue: Protein expression in Down syndrome brain. *J Neural Transm-Suppl* (61)
2002. Special issue: The Molecular Biology Database Collection - 2002 update. *Nucleic Acids Res* **30:** (1)
Aharoni A, Vorst O. 2002. Plant Res Int, Business Unit Cell Cybernet, POB 16, NL-6700 AA Wageningen, The Netherlands. DNA microarrays for functional plant genomics. *Plant Mol Biol* **48:** (1-2) 99.
Alam R, Gorska M. 2001. Univ Texas, Med Branch, Dept Internal Med, Div Allergy & Immunol, Galveston, Tx 77555, USA. Genomic microarrays: Arraying order in biological chaos? *Am J Respir Cell Mol Biol* **25:** (4) 405.
Bals R, Jany B. 2001. Hosp Univ Munich, Med Klin & Poliklin, Div Pulm, Dept Med, 1 Grosshadern, DE-81377 Munich, Germany. Identification of disease genes by expression profiling (Review). *Eur Resp J* **18:** (5) 882.
Bill RM. 2001. Gothenburg Univ, Dept Cell & Mol Biol Microbiol, Box 462, SE-40530 Gothenburg, Sweden. Yeast: A panacea for the structure-function analysis of membrane proteins? (Review). *Curr Genet* **40:** (3) 157.
Bubendorf L. 2001. Univ Basel, Inst Pathol, CH-4031 Basel, Switzerland. High-throughput microarray technologies: From genomics to clinics. *Eur Urol* **40:** (2) 231.
Buysse JM. 2001. Microcide Pharmaceut Inc, 850 Maude Ave, Mount View, Ca 94043, USA. The role of genomics in antibacterial target discovery. *Curr Med Chem* **8:** (14) 1713.
Castresana J. 2001. European Mol Biol Lab, Biocomp Unit, Meyerhofstr 1, DE-69117 Heidelberg, Germany. Comparative genomics and bioenergetics (Review). *Biochim Biophys Acta* **1506:** (3) 147.
Chen W, Tang DZ, Suo JF, Zhang YS, Xue YB*. 2001. *Chinese Acad Sci, Inst Dev Biol, Lab Plant Genet & Dev Biol, CN-100080 Beijing, Peoples Rep China. Expressional profiling of genes related to pollination and fertilization in rice. *C R Acad Sci Ser III-Vie* **324:** (12) 1111.
Choudhary JS, Blackstock WP, Creasy DM, Cottrell JS*. 2001. *GlaxoSmithKline R&D, Cell Mapping Project, Stevenage SG1 2NY, England. Matching peptide mass spectra to EST and genomic DNA databases (Review). *Trends Biotechnol* **19:** (10 Suppl) S17.
Colebatch G, Trevaskis B, Udvardi M*. 2002. *Max-Planck-Inst Mol Plant Physiol, Muhlenberg 1, DE-14476 Golm, Germany. Functional genomics: Tools of the trade (Review). *New Phytol* **153:** (1) 27.
Dacks JB, Doolittle WF*. 2001. *Dalhousie Univ, Dept Biochem & Mol Biol, Canadian Inst Adv Res, Program Evolutionary Biol, Halifax, Nova Scotia, Canada. Reconstructing/deconstructing the earliest eukaryotes: How comparative genomics can help (Review). *Cell* **107:** (4) 419.
Dean NM. 2001. GeneTrove, Dept Funct Genomics, 2292 Faraday Ave, Carlsbad, Ca 92008, USA. Functional genomics and target validation approaches using antisense oligonucleotide technology. *Curr Opin Biotechnol* **12:** (6) 622.
Dongre AR, Opiteck G, Cosand WL*, Hefta SA. 2001. *Bristol Myers Squibb Co, Pharmaceut Res Inst, Dept Appl Genomics, Princeton, NJ 08543, USA. Proteomics in the post-genome age. *Biopolymers* **60:** (3) 206.
Donson J, Fang YW, Espiritu-Santo G, Xing WM, Salazar A, Miyamoto S, Armendarez V, Volkmuth W. 2002. Ceres Inc, 3007 Malibu Canyon Rd, Malibu, Ca 90265, USA. Comprehensive gene expression analysis by transcript profiling. *Plant Mol Biol* **48:** (1-2) 75.
Engidawork E, Lubec G*. 2001. *Univ Vienna, Dept Pediat, Wahringer Gurtel 18-20, AU-1090 Vienna, Austria. Protein expression in Down syndrome brain (Review). *Amino Acids* **21:** (4) 331.
Famulok M, Blind M, Mayer G. 2001. Univ Bonn, Kekule Inst Organ Chem & Biochem, Gerhard Domagk Str 1, DE-53121 Bonn, Germany. Intramers as promising new tools in functional proteomics (Review). *Chem Biol* **8:** (10) 931.
Fiehn O. 2002. Max-Planck-Inst Mol Plant Physiol, DE-14424 Potsdam, Germany. Metabolomics: The link between genotypes and phenotypes. *Plant Mol Biol* **48:** (1-2) 155.
Finkelstein D, Ewing R, Gollub J, Sterky F, Cherry JM, Somerville S. 2002. Carnegie Inst Washington, Dept Plant Biol, 260 Panama St, Stanford, Ca 94305, USA. Microarray data quality analysis: Lessons from the AFGC project. *Plant Mol Biol* **48:** (1-2) 119.
Fountoulakis M. 2001. F Hoffmann La Roche & Co Ltd, Pharmaceut Res, Genomics Technol, Bldg 93-444, CH-4070 Basel, Switzerland. Proteomics: Current technologies and applications in neurological disorders and toxicology (Review). *Amino Acids* **21:** (4) 363.
Gabrielson E, Berg K, Anbazhagan R. 2001. Johns Hopkins Univ, Sch Med, Dept Pathol, 418 Nth Bond St, Baltimore, Md 21231, USA. Functional genomics, gene arrays, and the future of pathology (Review). *Mod Pathol* **14:** (12) 1294.
Goble C. 2001. Univ Manchester, Dept Comp Sci, Oxford Road, Manchester M13 9PL, England. The low down on e-science and grids for biology (Review). *Comp Funct Genom* **2:** (6) 365.
Grant SGN, Husi H. 2001. Univ Edinburgh, Dept Neurosci, 1 George Sq, Edinburgh EH8 9JZ, Scotland. Proteomics of multiprotein complexes: Answering fundamental questions in neuroscience (Review). *Trends Biotechnol* **19:** (10 Suppl) S49.
Griffin TJ, Aebersold R. 2001. Inst Syst Biol, 4225 Roosevelt Way Nth, Suite 200, Seattle, Wa 98105, USA. Advances in proteome analysis by mass spectrometry (Mini-Review). *J Biol Chem* **276:** (49) 45497.
Griffin TJ, Goodlett DR, Aebersold R. 2001. Address as above. Advances in proteome analysis by mass spectrometry. *Curr Opin Biotechnol* **12:** (6) 607.
Hanson IM. 2001. Univ Edinburgh, Western Gen Hosp, Dept Med Sci, Mol Med Ctr, Med Genet Sect, Crewe Rd, Edinburgh EH4 2XU, Scotland. Mammalian homologues of the *Drosophila* eye specification genes. *Semin Cell Dev Biol* **12:** (6) 475.
Herbert BR, Harry JL, Packer NH, Gooley AA, Pedersen SK, Williams KL. 2001. Proteome Syst, 1-35-41 Waterloo Rd, North Ryde, NSW 2113, Australia. What place for polyacrylamide in proteomics? (Review). *Trends Biotechnol* **19:** (10 Suppl) S3.
Herrmann PC, Liotta LA, Petricoin EF*. 2001. *US/FDA, Tissue Proteomics Unit, Div Therapeut Prod, Bldg 29A, Room 2802, 8800 Rockville Pike, Bethesda, Md 20892, USA. Cancer proteomics: The state of the art. *Dis Marker* **17:** (2) 49.
Ito T, Chiba T, Yoshida M. 2001. Kanazawa Univ, Canc Res Inst, Div Genome Biol, 13-1 Takaramachi, Kanazawa, Ishikawa 920 093, Japan. Exploring the protein interactome using comprehensive two-hybrid projects (Review). *Trends Biotechnol* **19:** (10 Suppl) S23.
Janson CG, During MJ. 2001. CNS Gene Therapy Ctr, 1025 Walnut St, Suite 511, Philadelphia, Pa 19107, USA. Viral vectors as part of an in-

In order to keep subscribers up-to-date with the latest developments in their field, this current awareness service is provided by John Wiley & Sons and contains newly-published material on comparative and functional genomics. Each bibliography is divided into 16 sections. 1 Reviews & symposia; 2 General; 3 Large-scale sequencing and mapping; 4 Evolutionary genomics; 5 Comparative genomics; 6 Pathways, gene families and regulons; 7 Pharmacogenomics; 8 EST, cDNA and other clone resources; 9 Functional genomics; 10 Transcriptomics; 11 Proteomics; 12 Protein structural genomics; 13 Metabolomics; 14 Genomic approaches to development; 15 Technological advances; 16 Bioinformatics. Within each section, articles are listed in alphabetical order with respect to author. If, in the preceding period, no publications are located relevant to any one of these headings, that section will be omitted.

Copyright © 2002 John Wiley & Sons, Ltd.

tegrated functional genomics program (Review). *Genomics* **78:** (1-2) 3.

Janson CG, McPhee SWJ, Leone P, Freese A, During MJ. 2001. Jefferson Med Coll, CNS Gene Therapy Ctr, 1025 Walnut St, Suite 511, Philadelphia, Pa 19107, USA. Viral-based gene transfer to the mammalian CNS for functional genomic studies (Review). *Trends Neurosci* **24:** (12) 706.

Jhoti H. 2001. Astex Technol, Cambridge CB4 0WE, England. High-throughput structural proteomics using x-rays (Review). *Trends Biotechnol* **19:** (10 Suppl) S67.

Jiang LL, Tsubakihara M, Heinke MY, Yao M, Dunn MJ, Phillips W, Dos Remedios CG*, Nosworthy NJ. 2001. *Univ Sydney, Dept Anat & Histol, Inst Biomed Res, Sydney, NSW 2006, Australia. Heart failure and apoptosis: Electrophoretic methods support data from micro- and macro-arrays. A critical review of genomics and proteomics (Review). *Proteomics* **1:** (12) 1481.

Kato-Maeda M, Gao Q, Small PM*. 2001. *Stanford Univ, Sch Med, Dept Med, Div Infect Dis & Geog Med, 300 Pasteur Dr, Grant Bldg, Stanford, Ca 94305, USA. Microarray analysis of pathogens and their interaction with hosts (Review). *Cell Microbiol* **3:** (11) 713.

Kellam P. 2001. UCL, Windeyer Inst Med Sci, Dept Immunol & Mol Pathol, Wohl Virion Ctr, 46 Cleveland St, London W1T 4JF, England. Post-genomic virology: The impact of bioinformatics, microarrays and proteomics on investigating host and pathogen interactions (Review). *Rev Med Virol* **11:** (5) 313.

Kersten B, Burkle L, Kuhn EJ, Giavalisco P, Konthur Z, Lueking A, Walter G, Eickhoff H, Schneider U. 2002. Max-Planck-Inst Mol Genet, Ihnestr 73, DE-14195 Berlin, Germany. Large-scale plant proteomics. *Plant Mol Biol* **48:** (1-2) 133.

Ko MSH. 2001. NIH/NIA, Dev Genome & Aging Sect, Genet Lab, Baltimore, Md 21224, USA. Embryogenomics: Developmental biology meets genomics (Review). *Trends Biotechnol* **19:** (12) 511.

Kornblum HL, Geschwind DH. 2001. UCLA, Sch Med, Dept Mol & Med Pharmacol, Room 1246, 700 Westwood Plaza, Los Angeles, Ca 90095, USA. The use of representational difference analysis and cDNA microarrays in neural repair research. *Restor Neurol Neurosci* **18:** (2-3) 89.

Kratz E, Dugas JC, Ngai J. 2002. Univ Calif Berkeley, Helen Wills Neurosci Inst, Dept Mol & Cell Biol, 269 Life Addit 3200, Berkeley, Ca 94720, USA. Odorant receptor gene regulation: Implications from genomic organization (Review). *Trends Genet* **18:** (1) 29.

Lorens JB, Sousa C, Bennett MK, Molineaux SM, Payan DG. 2001. Rigel Inc, 240 East Grand Ave, Sth San Francisco, Ca 94080, USA. The use of retroviruses as pharmaceutical tools for target discovery and validation in the field of functional genomics. *Curr Opin Biotechnol* **12:** (6) 613.

Macoska JA. 2002. Univ Michigan, Ctr Comprehens Canc, Dept Urol, Ann Arbor, Mi 48109, USA. The progressing clinical utility of DNA microarrays. *CA Cancer J Clin* **52:** (1) 50.

Maggio ET, Ramnarayan K. 2001. Struct Bioinformatics, San Diego, Ca 92127, USA. Recent developments in computational proteomics (Review). *Drug Discov Today* **6:** (19) 996.

Mahlknecht U, Ottmann OG, Hoelzer D. 2001. Univ Frankfurt, Dept Hematol Oncol, Med Ctr, Theodor Stern Kai 7, DE-60590 Frankfurt, Germany. Far-Western based protein-protein interaction screening of high-density protein filter arrays (Mini-Review). *J Biotechnol* **88:** (2) 89.

Maine EM. 2001. Syracuse Univ, Dept Biol, 108 Coll Pl, Syracuse, NY 13244, USA. RNAi as a tool for understanding germline development in *Caenohabditis elegans*: Uses and cautions (Review). *Dev Biol* **239:** (2) 177.

Marciano PG, Eberwine JH, Raghupathi R, McIntosh TK. 2001. Univ Penn, Dept Neurosurg, 105 Hayden Hall, 3320 Smith Walk, Philadelphia, Pa 19104, USA. The assessment of genomic alterations using DNA arrays following traumatic brain injury (Review). *Restor Neurol Neurosci* **18:** (2-3) 105.

Mehraban F, Tomlinson JE. 2001. Curagen Corp, 555 Long Wharf Dr, New Haven, Ct 06511, USA. Application of industrial scale genomics to discovery of therapeutic targets in heart failure (Review). *Eur J Heart Fail* **3:** (6) 641.

Mills DA. 2001. Univ Calif Davis, Dept Viticulture & Enol, 1 Shields Ave, Davis, Ca 95616, USA. Mutagenesis in the post genomics era: Tools for generating insertional mutations in the lactic acid. *Curr Opin Biotechnol* **12:** (5) 503.

Mitchell-Olds T, Clauss MJ. 2002. Max-Planck-Inst Chem Ecol, Dept Genet & Evolut, DE-07745 Jena, Germany. Plant evolutionary genomics. *Curr Opin Plant Biol* **5:** (1) 74.

Moseley MA. 2001. GlaxoSmithKline, Genet Res, POB 13398, Res Triangle Pk, NC 27709, USA. Current trends in differential expression proteomics: Isotopically coded tags (Review). *Trends Biotechnol* **19:** (10 Suppl) S10.

Nakanishi T, Oka T, Akagi T. 2001. Okayama Univ, Grad Sch Med & Dent, Dept Biochem & Mol Dent, Okayama 700 8525, Japan. Recent advances in DNA microarrays (Review). *Acta Med Okayama* **55:** (6) 319.

Natsume T, Nakayama H, Isobe T. 2001. Natl Inst Adv Sci & Technol, JBIRC, Biol Informat Res Ctr, Kohtoh ku, 2-41-6 Ohmi, Tokyo 135 0064, Japan. BIA-MS-MS: Biomolecular interaction analysis for functional proteomics (Review). *Trends Biotechnol* **19:** (10 Suppl) S28.

Orengo CA, Bray JE, Buchan DWA, Harrison A, Lee D, Pearl FMG, Sillitoe I, Todd AE, Thornton JM. 2002. UCL, Dept Biochem & Mol Biol, Gower St, London WC1E 6BT, England. The CATH protein family database: A resource for structural and functional annotation of genomes - Review. *Proteomics* **2:** (1) 11.

Pertea M, Salzberg SL. 2002. Inst Genome Res, 9712 Med Ctr Dr, Rockville, Md 20850, USA. Computational gene finding in plants. *Plant Mol Biol* **48:** (1-2) 39.

Phillips KA, Veenstra DL, Oren E, Lee JK, Sadee W. 2001. Univ Calif, Inst Hlth Policy Studies, Sch Pharm, Dept Clin Pharm, 3333 Calif St, San Francisco, Ca 94143, USA. Potential role of pharmacogenomics in reducing adverse drug reactions: A systematic review. *JAMA* **286:** (18) 2270.

Prozorov AA. 2001. Russian Acad Sci, Vavilov Inst Gen Genet, Moscow, Russia. Recombinational rearrangements in bacterial genome and bacterial adaptation to the environment (Review). *Microbiology-Engl Tr* **70:** (5) 501.

Puig O, Caspary F, Rigaut G, Rutz B, Bouveret E, Bragado-Nilsson E, Wilm M, Seraphin B*. 2001. *CNRS CGM, Ave Terrasse, FR-91198 Gif-sur-Yvette, France. The tandem affinity purification (TAP) method: A general procedure of protein complex purification (Review). *Methods* **24:** (3) 218.

Rabilloud T. 2002. CEA, DBMS BECP, Lab Bioenerget Cellulaire & Pathol, 17 rue Martyrs, FR-38054 Grenoble 9, France. Two-dimensional gel electrophoresis in proteomics: Old, old fashioned, but it still climbs up the mountains - Review. *Proteomics* **2:** (1) 3.

Raetz EA, Moos PJ, Szabo A, Carroll WL*. 2001. *CUNY Mt Sinai Sch Med, Combined Program Pediat Hematol Oncol, Box 1308, 1 Gustave Levy Pl, New York, NY 10029, USA. Gene expression profiling: Methods and clinical applications in oncology. *Hematol Oncol Clin North Am* **15:** (5) 911.

Regnier FE, Riggs L, Zhang R, Xiong L, Liu P, Chakraborty A, Seeley E, Sioma C, Thompson RA. 2002. Purdue Univ, Dept Chem, West Lafayette, In 47907, USA. Special feature: Perspective - Comparative proteomics based on stable isotope labeling and affinity selection. *J Mass Spectrom* **37:** (2) 133.

Rehm BHA. 2001. Univ Munster, Inst Mikrobiol, Corrensstr 3, DE-48149 Munster, Germany. Bioinformatic tools for DNA/protein sequence analysis, functional assignment of genes and protein classification (Mini-Review). *Appl Microbiol Biotechnol* **57:** (5-6) 579.

Reiser L, Mueller LA, Rhee SY. 2002. Carnegie Inst Washington, Dept Plant Biol, 260 Panama St, Stanford, Ca 94305, USA. Surviving in a sea of data: A survey of plant genome data resources and issues in building data management systems. *Plant Mol Biol* **48:** (1-2) 59.

Roberts JKM. 2002. Univ Calif, Dept Biochem, Riverside, Ca 92521, USA. Proteomics and a future generation of plant molecular biologists. *Plant Mol Biol* **48:** (1-2) 143.

Schmidt R. 2002. Max-Planck-Inst Mol Pflanzenphysiol, Muhlenberg 1, DE-14476 Golm, Germany. Plant genome evolution: Lessons from comparative genomics at the DNA level. *Plant Mol Biol* **48:** (1-2) 21.

Schrader M, Schulz-Knappe P. 2001. BioVisioN GmbH & Co KG, Feodor Lynen Str 5, DE-30625 Hannover, Germany. Peptidomics technologies for human body fluids (Review). *Trends Biotechnol* **19:** (10 Suppl) S55.

Simpson RJ, Dorow DS. 2001. Ludwig Inst Canc Res, Walter & Eliza Hall Inst Med Res, Joint Prot Struct Lab, Parkville, Vic 3050, Australia. Cancer proteomics: From signaling networks to tumor markers (Review). *Trends Biotechnol* **19:** (10 Suppl) S40.

Turecek F. 2002. Univ Washington, Dept Chem, Bagley Hall, Box 351700, Seattle, Wa 98195, USA. Special feature: Perspective - Mass spectrometry in coupling with affinity capture-release and isotope-coded affinity tags for quantitative protein analysis. *J Mass*

Copyright © 2002 John Wiley & Sons, Ltd.

*Spectrom* **37:** (1) 1.

Turkewitz AP, Orias E, Kapler G. 2002. Univ Chicago, Dept Mol Genet & Cell Biol, 920 East 58th St, Chicago, Il 60637, USA. Functional genomics: The coming of age for *Tetrahymena thermophila* (Review). *Trends Genet* **18:** (1) 35.

Van der Vet P, Roosendaal HE, Geurts PATM. 2001. Univ Twente, Dept Comput Sci, POB 217, NL-7500 AE Enschede, The Netherlands. $C_2M$: Configurable chemical middleware (Review). *Comp Funct Genom* **2:** (6) 371.

Walke DW, Han CS, Shaw J, Wann E, Zambrowicz B, Sands A. 2001. Lexicon Genet Inc, 4000 Res Forest Dr, The Woodlands, Tx 77381, USA. *In vivo* drug target discovery: Identifying the best targets from the genome. *Curr Opin Biotechnol* **12:** (6) 626.

Weir M, Swindells M, Overington J. 2001. Inpharmatica, 60 Charlotte St, London W1T 2NU, England. Insights into protein function through large-scale computational analysis of sequence and structure (Review). *Trends Biotechnol* **19:** (10 Suppl) S61.

Wu SH, Ramonell K, Gollub J, Somerville S. 2001. Carnegie Inst Washington, Dept Plant Biol, 260 Panama St, Stanford, Ca 94305, USA. Plant gene expression profiling with DNA microarrays (Review). *Plant Physiol Biochem* **39:** (11) 917.

Ye RW, Wang T, Bedzyk L, Croker KM. 2001. DuPont Co Inc, Cent Res & Dev, DuPont Expt Stn, Route 141 & Henry Clay Rd, Wilmington, De 19880, USA. Applications of DNA microarrays in microbial systems (Review). *J Microbiol Methods* **47:** (3) 257.

Zhou HH, Roy S, Schulman H, Natan MJ*. 2001. *SurroMed, 2375 Garcia Ave, Mount View, Ca 94043, USA. Solution and chips arrays in protein profiling (Review). *Trends Biotechnol* **19:** (10 Suppl) S34.

### 3 Large-scale sequencing and mapping

Barakat A, Szick-Miranda K, Chang IF, Guyot R, Blanc G, Cooke R, Delseny M, Bailey-Serres J*. 2001. *Univ Calif, Dept Bot & Plant Biol, Riverside, Ca 92521, USA. The organization of cytoplasmic ribosomal protein genes in the *Arabidopsis* genome. *Plant Physiol* **127:** (2) 398.

Fitz-Gibbon ST, Ladner H, Kim UJ, Stetter KO, Simon MI, Miller JH*. 2002. *UCLA, Dept Microbiol Immunol & Mol Genet, Los Angeles, Ca 90095, USA. Genome sequence of the hyperthermophilic crenarchaeon *Pyrobaculum aerophilum*. *Proc Natl Acad Sci U S A* **99:** (2) 984.

Goodner B, Hinkle G, Gattung S, Miller N, Blanchard M, Qurollo B, Goldman BS, Cao Y, Askenazi M, Halling C *et al.* 2001. c/o Slater S, Cereon Genomics LLC, 45 Sydney St, Cambridge, Ma 02139, USA. Genome sequence of the plant pathogen and biotechnology agent *Agrobacterium tumefaciens* C58. *Science* **294:** (5550) 2323.

Kaneko T, Nakamura Y, Wolk C P, Kuritz T, Sasamoto S, Watanabe A, Iriguchi M, Ishikawa A, Kawashima K, Kimura T *et al.* 2001. c/o Tabata S, Kazusa DNA Res Inst, 153203 Yana, Chiba 292 0812, Japan. Complete genomic sequence of the filamentous nitrogen-fixing cyanobacterium *Anabaena* sp strain PCC 7120. *DNA Res* **8:** (5) 205.

McClelland M, Sanderson KE, Spieth J, Clifton SW, Latreille P, Courtney L, Porwollik S, Ali J, Dante M, Du F *et al.* 2001. Sidney Kimmel Canc Ctr, 10835 Altman Row, San Diego, Ca 92121, USA. Complete genome sequence of *Salmonella enterica* serovar *typhimurium* LT2. *Nature* **413:** (6858) 852.

Myler PJ, Beverley SM, Cruz AK, Dobson DE, Ivens AC, McDonagh PD, Madhubala R, Martinez-Calvillo S, Ruiz JC, Saxena A *et al.* 2001. Seattle Biomed Res Inst, 4 Nickerson St, Seattle, Wa 98109, USA. The Leishmania genome project: New insights into gene organization and function. *Med Microbiol Immunol* **190:** (1-2) 9.

Parkhill J, Dougan G, James KD, Thomson NR, Pickard D, Wain J, Churcher C, Mungall KL, Bentley SD, Holden MT *et al.* 2001. Sanger Ctr, Wellcome Trust Genome Campus, Cambridge CB10 1SA, England. Complete genome sequence of a multiple resistant *Salmonella enterica* serovar *typhi* CT18. *Nature* **413:** (6858) 848.

Shimizu T, Ohtani K, Hirakawa H, Oshima K, Yamashita A, Shiba T, Ogasawara N, Hattori M, Kuhara S, Hayashi H. 2002. Univ Tsukuba, Inst Basic Med Sci, Dept Microbiol, Tsukuba, Ibaraki 305 8575, Japan. Complete genome sequence of *Clostridium perfringens*, an anaerobic flesh-eater. *Proc Natl Acad Sci U S A* **99:** (2) 996.

Wood DW, Setubal JC, Kaul R, Monks DE, Kitajima JP, Okura VK, Zhou Y, Chen L, Wood GE, Almeida NF *et al.* 2001. c/o Nester EW, Univ Washington, Dept Microbiol, 1959 NE Pacific St, Box 357242, Seattle, Wa 98195, USA. The genome of the natural genetic engineer *Agrobacterium tumefaciens* C58. *Science* **294:** (5550) 2317.

### 4 Evolutionary genomics

Bourque G, Pevzner PA. 2002. Univ Sthn Calif, Dept Math, Los Angeles, Ca 90089, USA. Genome-scale evolution: Reconstructing gene orders in the ancestral species. *Genome Res* **12:** (1) 26.

Broughton RE, Milam JE, Roe BA. 2001. Univ Oklahoma, Oklahoma Biol Survey, Norman, Ok 73019, USA. The complete sequence of the zebrafish (*Danio rerio*) mitochondrial genome and evolutionary patterns in vertebrate mitochondrial DNA. *Genome Res* **11:** (11) 1958.

Fischer G, Neuveglise C, Durrens P, Gaillardin C, Dujon B. 2001. Univ Paris 06, CNRS, Unite Genet Mol Levures, Inst Pasteur, FR-75724 Paris 15, France. Evolution of gene order in the genomes of two related yeast species. *Genome Res* **11:** (12) 2009.

Kuhn G, Hijri M, Sanders IR*. 2001. *Univ Lausanne, Inst Ecol, Biol Bldg, CH-1015 Lausanne, Switzerland. Evidence for the evolution of multiple genomes in arbuscular mycorrhizal fungi. *Nature* **414:** (6865) 745.

Qian J, Luscombe NM, Gerstein M*. 2001. *Yale Univ, Dept Mol Biophys & Biochem, 266 Whitney Ave, POB 208114, New Haven, Ct 06520, USA. Protein family and fold occurrence in genomes: Power-law behaviour and evolutionary model. *J Mol Biol* **313:** (4) 673.

Richly E, Kurth J, Leister D*. 2002. *Max-Planck-Inst Zuchtungsforsch, Abt Pflanzenzuchtung & Ertragsphysiol, Carl von Linne Weg 10, DE-50829 Cologne, Germany. Mode of amplification and reorganization of resistance genes during recent *Arabidopsis thaliana* evolution. *Mol Biol Evol* **19:** (1) 76.

Salemi M, Vandamme AM. 2002. Katholieke Univ Leuven, Rega Inst Med Res, Minderbroedersstr 10, BE-3000 Louvain, Belgium. Hepatitis C virus evolutionary patterns studied through analysis of full-genome sequences. *J Mol Evol* **54:** (1) 62.

Snel B, Bork P, Huynen MA. 2002. European Mol Biol Lab, DE-69117 Heidelberg, Germany. Genomes in flux: The evolution of archaeal and proteobacterial gene content. *Genome Res* **12:** (1) 17.

### 5 Comparative genomics

Bolshakov VN, Topalis P, Blass C, Kokoza E, Della'Torre A, Kafatos FC, Louis C*. 2002. *FORTH, Inst Mol Biol & Biotechnol, Genome Res Lab, GR-71110 Iraklion, Crete, Greece. A comparative genomic analysis of two distant Diptera, the fruit fly, *Drosophila melanogaster*, and the malaria mosquito, *Anopheles gambiae*. *Genome Res* **12:** (1) 57.

Chen R, Bouck JB, Weinstock GM, Gibbs RA. 2001. Baylor Coll Med, Human Genome Sequencing Ctr, Dept Mol & Human Genet, Houston, Tx 77030, USA. Comparing vertebrate whole-genome shotgun reads to the human genome. *Genome Res* **11:** (11) 1807.

Ge ZM, Feng Y, White DA, Schauer DB, Fox JG. 2001. MIT, Div Comparat Med, 77 Massachusetts Ave, Cambridge, Ma 02139, USA. Genomic characterization of *Helicobacter hepaticus*: Ordered cosmid library and comparative sequence analysis. *FEMS Microbiol Lett* **204:** (1) 147.

Liu H, Sachidanadam R, Stein L*. 2001. *Cold Spring Harbor Lab, POB 100, Cold Spring Harbor, NY 11724, USA. Comparative genomics between rice and *Arabidopsis* shows scant collinearity in gene order. *Genome Res* **11:** (12) 2020.

### 6 Pathways, gene families and regulons

Bonanno JB, Edo C, Eswar N, Pieper U, Romanowski MJ, Ilyin V, Gerchman SE, Kycia H, Studier FW, Sali A, Burley SK*. 2001. *Rockefeller Univ, Labs Mol Biophys, 1230 York Ave, New York, NY 10021, USA. Structural genomics of enzymes involved in sterol/isoprenoid biosynthesis. *Proc Natl Acad Sci U S A* **98:** (23) 12896.

Doerks T, Copley RR, Schultz J, Ponting CP, Bork P. 2002. European Mol Biol Lab, Postfach 102209, Meyerhofstr 1, DE-69114 Heidelberg, Germany. Systematic identification of novel protein domain families associated with nuclear functions. *Genome Res* **12:** (1) 47.

Evertsz EM, Au-Young J, Ruvolo MV, Lim AC, Reynolds MA*. 2001. *Incyte Genomics, 3160 Porter Ave, Palo Alto, Ca 94304, USA. Hy-

Copyright © 2002 John Wiley & Sons, Ltd. 

bridization cross-reactivity within homologous gene families on glass cDNA microarrays. *Biotechniques* **31:** (5) 1182.

Jackson RM, Russell RB. 2001. UCL, Dept Biochem & Mol Biol, Gower St, London WC1E 6BT, England. Predicting function from structure: Examples of the serine protease inhibitor canonical loop conformation found in extracellular proteins. *Comput Chem* **26:** (1) 31.

Li XM, Novotna J, Vohradsky J, Weiser J*. 2001. *Acad Sci Czech Republ, Inst Microbiol, Videnska 1083, CZ-14220 Prague 4, Czech Republic. Major proteins related to chlortetracycline biosynthesis in a *Streptomyces aureofaciens* production strain studied by quantitative proteomics. *Appl Microbiol Biotechnol* **57:** (5-6) 717.

Van Rossum AJ, Brophy PM, Tait A, Barrett J, Jefferies JR. 2001. Univ Wales, Inst Biol Sci, Edward Llwyd Bldg, Aberystwyth SY23 3DS, Wales. Proteomic identification of glutathione S-transferases from the model nematode *Caenorhabditis elegans*. *Proteomics* **1:** (11) 1463.

Yanagida M, Shimamoto A, Nishikawa K, Furuichi Y, Isobe T, Takahashi N*. 2001. *Tokyo Univ Agr & Technol, Dept Appl Biol Sci, 3-5-8 Saiwai cho, Tokyo 183 8509, Japan. Isolation and proteomic characterization of the major proteins of the nucleolin-binding ribonucleoprotein complexes. *Proteomics* **1:** (11) 1390.

### 7 Pharmacogenomics

Ahr A, Karn T, Solbach C, Seiter T, Strebhardt K, Holtrich U, Kaufmann M. 2002. Univ Frankfurt, Dept Obstet & Gynecol, Theodor Stern Kai 7, DE-60590 Frankfurt, Germany. Identification of high risk breast-cancer patients by gene expression profiling. *Lancet* **359:** (9301) 131.

Baranova AV, Lobashev AV, Ivanova DV, Krukovskaya LL, Yankovsky NK, Kozlov AP*. 2001. *Res Inst Pure Biochem, Biomed Ctr, 7 Pudozhskaya Str, RU-197110 St Petersburg, Russia. *In silico* screening for tumour-specific expressed sequences in human genome. *FEBS Lett* **508:** (1) 143.

Bezchlibnyk YB, Wang JF, McQueen GM, Young LT*. 2001. *McMaster Univ, Dept Psychiat & Behav Neurosci, 1200 Main St West, Hamilton, Ontario, Canada L8N 3Z5. Gene expression differences in bipolar disorder revealed by cDNA array analysis of post-mortem frontal cortex. *J Neurochem* **79:** (4) 826.

Blakely RD. 2001. Vanderbilt Univ, Sch Med, Ctr Mol Neurosci, Dept Pharmacol, 417 Preston Res Bldg, Nashville, Tn 37232, USA. Physiological genomics of antidepressant targets: Keeping the periphery in mind. *J Neurosci* **21:** (21) 8319.

Boer JM, Huber WK, Sultmann H, Wilmer F, Von Heydebreck A, Haas S, Korn B, Gunawan B, Vente A, Fuzesi L *et al*. 2001. c/o Poustka A, German Canc Res Ctr, Dept Mol Genome Anal, DE-6900 Heidelberg, Germany. Identification and classification of differentially expressed genes in renal cell carcinoma by expression profiling on a global human 31,5000-element cDNA array. *Genome Res* **11:** (11) 1861.

Caballero OL, De Souza SJ, Brentani RR, Simpson AJG*. 2001. *Ludwig Inst Canc Res, Rua Prof Antonio Prudente 109-40 Andar, BR-01509-010 Sao Paulo, SP, Brazil. Alternative spliced transcripts as cancer markers. *Dis Marker* **17:** (2) 67.

Chen YD, Yakhini Z, Ben Dor A, Dougherty E, Trent JM, Bittner M*. 2001. *NIH/NHGRI, Canc Genet Branch, Bldg 49, Room 4A52, 9000 Rockville Pike, Rockville, Md 20850, USA. Analysis of expression patterns: The scope of the problem, the problem of scope. *Dis Marker* **17:** (2) 59.

Cole J, Tsou R, Wallace K, Gibran N, Isik F. 2001. Univ Washington, Dept Surg, Med Ctr, Box 356410, 1959 NE Pacific St, Seattle, Wa 98195, USA. Early gene expression profile of human skin to injury using high-density cDNA microarrays. *Wound Repair Regen* **9:** (5) 360.

Cortesini NSF, Piazza F, Ho E, Ciubotariu R, Le Maoult J, Dalla Favera R, Cortesini R. 2001. Columbia Univ, Dept Pathol, 630 West 168th St, New York, NY 10032, USA. Distinct mRNA microarray profiles of tolerogenic dendritic cells. *Hum Immunol* **62:** (10) 1065.

Cox JM, Clayton CL, Tomita T, Wallace DM, Robinson PA, Crabtree JE*. 2001. *St James Univ Hosp, Mol Med Unit, Level 7, Clin Sci Bldg, Leeds LS9 7TF, England. cDNA array analysis of *cag* pathogenicity island-associated *Helicobacter pylori* epithelial cell response genes. *Infect Immun* **69:** (11) 6970.

Friess H, Ding JY, Kleeff J, Liao Q, Berberat PO, Hammer J, Buchler MW. 2001. Univ Heidelberg, Dept Gen Surg, Neuenheimer Feld 110, DE-69120 Heidelberg, Germany. Identification of disease-specific genes in chronic pancreatitis using DNA array technology. *Ann Surg* **234:** (6) 769.

Fuller GN, Hess KR, Rhee CH, Yung WKA, Sawaya RA, Bruner JM, Zhang W. 2002. Univ Texas, MD Anderson Canc Ctr, Dept Pathol, Houston, Tx 77030, USA. Molecular classification of human diffuse gliomas by multidimensional scaling analysis of gene expression profiles parallels morphology-based classification, correlates with survival, and reveals clinically-relevant novel glioma subsets. *Brain Pathol* **12:** (1) 108.

Gebauer M, Von Melchner H*, Beckers T. 2001. *Univ Frankfurt, Sch Med, Lab Mol Hematol, DE-6000 Frankfurt, Germany. Genomewide trapping of genes that encode secreted and transmembrane proteins repressed by oncogenic signaling. *Genome Res* **11:** (11) 1871.

Guillonneau F, Labas V, Auvin C, Praseuth D*. 2001. *Museum Nal Hist Nat, CNRS UMR 8646 INSERM U201, Biophys Lab, 43 rue Cuvier, FR-75231 Paris 05, France. A reliable and simple method for two-dimensional electrophoresis and identification of HeLa nuclear alkaline nucleic acid-binding proteins using immobilized pH gradient. *Electrophoresis* **22:** (20) 4391.

Hsiao LL, Dangond F, Yoshida T, Hong R, Jensen RV, Misra J, Dillon W, Lee KF, Clark KE, Haverty P *et al*. 2001. c/o Gullans SR, 65 Landsdowne, Cambridge, Ma 02140, USA. A compendium of gene expression in normal human tissues. *Physiol Genomics* **7:** (2) 97.

Hurko O. 2001. GlaxoSmithKline, Neurol Ctr Excellence Drug Discovery New Frontier, Harlow CM19 5AW, England. Genetics and genomics in neuropsychopharmacology: The impact on drug discovery and development. *Eur Neuropsychopharmacol* **11:** (6) 491.

Jhaveri MS, Wagner C, Trepel JB. 2001. Georgetown Univ, Dept Oncol, Med Ctr, Vincent T Lombardi Canc Res Ctr, Washington, DC 20007, USA. Impact of extracellular folate levels on global gene expression. *Mol Pharmacol* **60:** (6) 1288.

Joachimiak MP, Chang C, Rosenthal PJ, Cohen FE*. 2001. *Univ Calif, Dept Cellular & Mol Pharmacol, Box 0450, San Francisco, Ca 94143, USA. The impact of whole genome sequence data on drug discovery: A malaria case study. *Mol Med* **7:** (10) 698.

Jun AS, Liu SH, Koo EH, Do DV, Stark WJ, Gottsch JD*. 2001. *Johns Hopkins Univ Hosp, Inst Med, Wilmer Ophthalmol Inst, Cornea & External Dis Div, Maumenee 317, Baltimore, Md 21287, USA. Microarray analysis of gene expression in human donor corneas. *Arch Ophthalmol* **119:** (11) 1629.

Katsuma S, Nishi K, Tanigawara K, Ikawa H, Shiojima S, Takagaki K, Kaminishi Y, Suzuki Y, Hirasawa A, Ohgi T *et al*. 2001. c/o Tsujimoto G, Natl Childrens Med Res Ctr, Dept Mol Cell Pharmacol, Setagaya ku, 3-35-31 Taishido, Tokyo 154 8509, Japan. Molecular monitoring of bleomycin-induced pulmonary fibrosis by cDNA microarray-based gene expression profiling. *Biochem Biophys Res Commun* **288:** (4) 747.

Locklin RM, Riggs BL, Hicok KC, Horton HF, Byrne MC, Khosla S*. 2001. *Mayo Clin & Mayo Fdn, Endocrine Res Unit, 200 1st St SW, Rochester, Mn 55905, USA. Assessment of gene regulation by bone morphogenetic protein 2 in human marrow stromal cells using gene array technology. *J Bone Miner Res* **16:** (12) 2192.

Loring JF, Wen X, Lee JM, Seilhamer J, Somogyi R. 2001. Incyte Genomics, Dept Life Sci, 3160 Porter Dr, Palo Alto, Ca 94304, USA. A gene expression profile of Alzheimer's disease. *DNA Cell Biol* **20:** (11) 683.

Los G, Yang F, Samimi G, Manorek G, Guerorguieva IM, Howell S, Van Erp N, Breaux JK. 2002. Univ Calif San Diego, Canc Ctr, La Jolla, Ca 92037, USA. Using mRNA expression profiling to determine anticancer drug efficacy. *Cytometry* **47:** (1) 66.

Manderson EN, Mes-Masson AM, Novak J, Lee PD, Provencher D, Hudson TJ, Tonin PN*. 2002. *McGill Univ, Dept Human Genet, Montreal, Quebec, Canada H3A 1B1. Expression profiles of 290 ESTs mapped to chromosome 3 in human epithelial ovarian cancer cell lines using DNA expression oligonucleotide microarrays. *Genome Res* **12:** (1) 112.

Matejuk A, Dwyer J, Zamora A, Vandenbark AA, Offner H. 2002. Vet Affairs Med Ctr, 3710 SW US Vet Hosp Rd, Portland, Or 97201, USA. Evaluation of the effects of 17β-estradiol (17β-E2) on gene expression in experimental autoimmune encephalomyelitis using DNA microarray. *Endocrinology* **143:** (1) 313.

Nakeff A, Sahay N, Pisano M, Subramanian B. 2002. Henry Ford Hlth Syst, Josephine Ford Canc Ctr, Drug Discovery & Dev Program, 1 Ford Pl 1D, Detroit, Mi 48202, USA. Painting with a molecular brush: Genomic/proteomic interfacing to define the drug action profile of novel solid-tumor selective anticancer agents. *Cytometry* **47:** (1) 72.

Copyright © 2002 John Wiley & Sons, Ltd. 

Ngimbous BB, Bourgeois F, Mas C, Simonneau M, Moalic JM*. 2001. *Hop Lariboisiere, INSERM U127, 41 Blvd Chapelle, FR-75475 Paris 10, France. Heart transplantation changes the expression of distinct gene families. *Physiol Genomics* **7:** (2) 115.

Oriola J, Halperin I, Mallofre C, Muntane J, Angel M, Rivera-Fillat F. 2001. Hosp Clin & Univ, IDIBAPS, Serv Hormonol, Barcelona, Spain. Screening of selected genomic areas potentially involved in thyroid neoplasms. *Eur J Cancer* **37:** (18) 2470.

Park KS, Cho SY, Kim H, Paik YK*. 2002. *Yonsei Univ, Dept Biochem, Yonsei Proteome Res Ctr, S309 Kwahak won, 134 Shinchon dong Sudaemoon ku, Seoul 120749, South Korea. Proteomic alterations of the variants of human aldehyde dehydrogenase isozymes correlate with hepatocellular carcinoma. *Int J Cancer* **97:** (2) 261.

Pasinetti GM, Ho L. 2001. Mt Sinai Sch Med, Dept Psychiat, Neuroinflammat Res Labs, 1 Gustave L Levy Pl, New York, NY 10029, USA. From cDNA microarrays to high-throughput proteomics. Implications in the search for preventive initiatives to slow the clinical progression of Alzheimer's disease dementia. *Restor Neurol Neurosci* **18:** (2-3) 137.

Poon TCW, Johnson PJ*. 2001. *Chinese Univ, Prince of Wales Hosp, Sir YK Pao Canc Ctr, Dept Clin Oncol, Shatin, Hong Kong, Peoples Rep China. Proteome analysis and its impact on the discovery of serological tumor markers. *Clin Chim Acta* **313:** (1-2) 231.

Rae JM, Johnson MD, Lippman ME, Flockhart DA. 2001. Univ Michigan, Dept Internal Med, Room 5312, 1500 East Med Ctr Dr, Ann Arbor, Mi 48109, USA. Rifampin is a selective, pleiotropic inducer of drug metabolism genes in human hepatocytes: Studies with cDNA and oligonucleotide expression arrays. *J Pharmacol Exp Ther* **299:** (3) 849.

Ramaswamy S, Tamayo P, Rifkin R, Mukherjee S, Yeang CH, Angelo M, Ladd C, Reich M, Latulippe E, Mesirov JP *et al*. 2001. c/o Golub TR, Harvard Univ, Sch Med, Dept Adult Oncol, Dana Farber Canc Inst, 44 Binney St, Boston, Ma 02115, USA. Multiclass cancer diagnosis using tumor gene expression signatures. *Proc Natl Acad Sci U S A* **98:** (26) 15149.

Rigolet M, Faussillon M, Baudry D, Junien C, Jeanpierre C. 2001. Univ Paris 05, Hop Necker Enfants Malad, U383 INSERM, 149 rue Sevres, FR-75015 Paris, France. Profiling of differential gene expression in Wilms tumor by cDNA expression array. *Pediatr Nephrol* **16:** (12) 1113.

Rooney PH, Boonsong A, McKay JA, Marsh S, Stevenson DAJ, Murray GI, Curran S, Haites NE, Cassidy K, McLeod HL*. 2001. *Washington Univ, Sch Med, Dept Med, 660 Sth Euclid Ave, Campus Box 8069, St Louis, Mo 63110, USA. Colorectal cancer genomics: Evidence for multiple genotypes which influence survival. *Br J Cancer* **85:** (10) 1492.

Rosenberger CM, Pollard AJ, Finlay BB*. 2001. *Univ British Columbia, Biotechnol Lab, 6174 Univ Blvd, Vancouver, Brit Columbia, Canada V6T 1Z3. Gene array technology to determine host responses to *Salmonella*. *Microbes Infect* **3:** (14-15) 1353.

Ross DT, Perou CM*. 2001. *Univ Nth Carolina, Lineberger Comprehens Canc Ctr, CB 7295, Chapel Hill, NC 27599, USA. A comparison of gene expression signatures from breast tumors and breast tissue derived cell lines. *Dis Marker* **17:** (2) 99.

Rybicki BA, Iyengar SK, Harris T, Liptak R, Elston RC, Maliarik MJ, Iannuzzi MC. 2002. Henry Ford Hlth Syst, Dept Biostat & Res Epidemiol, 1 Ford Pl, Detroit, Mi 48202, USA. Prospects of admixture linkage disequilibrium mapping in the African-American genome. *Cytometry* **47:** (1) 63.

Schonberger SJ, Edgar PF, Kydd R, Faull RLM, Cooper GJS*. 2001. *Univ Auckland, Sch Biol Sci, Private Bag 92019, Auckland 1, New Zealand. Proteomic analysis of the brain in Alzheimer's disease: Molecular phenotype of a complex disease process. *Proteomics* **1:** (12) 1519.

Schwartz JC, Morisset S, Rouleau A, Tardivel-Lacombe J, Gbahou F, Ligneau X, Heron A, Sasse A, Stark H, Schunack W *et al*. 2001. INSERM U109, Unite Neurobiol & Pharmacol Mol, Ctr Paul Broca, 2ter rue Alesia, FR-75014 Paris, France. Application of genomics to drug design: The example of the histamine $H_3$ receptor. *Eur Neuropsychopharmacol* **11:** (6) 441.

Sibille E, Hen R*. 2001. *Columbia Univ, Ctr Neurobiol & Behav, 722 West 168th St, New York, NY 10032, USA. Combining genetic and genomic approaches to study mood disorders. *Eur Neuropsychopharmacol* **11:** (6) 413.

Smith DI. 2002. Mayo Clin, Canc Genet Program, Canc Ctr, Rochester, Mn 55905, USA. Transcriptional profiling develops molecular signatures for ovarian tumors. *Cytometry* **47:** (1) 60.

Song GQ, Cechvala C, Resnick DK, Dempsey RJ, Rao VLR*. 2001. *Univ Wisconsin, Dept Neurol Surg, 600 Highland Ave, Madison, Wi 53792, USA. GeneChip® analysis after acute spinal cord injury in rat. *J Neurochem* **79:** (4) 804.

Steel LF, Mattu TS, Mehta A, Hebestreit H, Dwek R, Evans AA, London WT, Block T*. 2001. *Thomas Jefferson Univ, Jefferson Ctr Biomed Res, Dept Biochem & Mol Pharmacol, Doylestown, Pa 18901, USA. A proteomic approach for the discovery of early detection markers of hepatocellular carcinoma. *Dis Marker* **17:** (3) 179.

Sugiyama Y, Sugiyama K, Hirai Y, Akiyama F, Hasumi K. 2002. Canc Inst Hosp, Dept Gynecol, Toshima ku, 1-37-1 Kami Ikebukuro, Tokyo 170 8455, Japan. Microdissection is essential for gene expression profiling of clinically resected cancer tissues. *Am J Clin Pathol* **117:** (1) 109.

Thomas RS, Rank DR, Penn SG, Zastrow GM, Hayes KR, Pande K, Glover E, Silander T, Craven MW, Reddy JK, Jovanovich SB, Bradfield CA*. 2001. *Univ Wisconsin, Sch Med, McArdle Lab Canc Res, 1400 Univ Ave, Madison, Wi 53706, USA. Identification of toxicologically predictive gene sets using cDNA microarrays. *Mol Pharmacol* **60:** (6) 1189.

Valerio A, Basso D, Mazza S, Baldo G, Tiengo A, Pedrazzoli S, Seraglia R, Plebani M. 2001. Univ Padova, Dipt Med Clin & Sperimentale, via Giustiniani 2, IT-35128 Padova, Italy. Serum protein profiles of patients with pancreatic cancer and chronic pancreatitis: Searching for a diagnostic protein pattern. *Rapid Commun Mass Spectrom* **15:** (24) 2420.

Valery C, Grob JJ, Verrando P*. 2001. *Univ Mediterranee, LIMP, 46 Blvd Gaye, FR-13009 Marseille, France. Identification by cDNA microarray technology of genes modulated by artificial ultraviolet radiation in normal human melanocytes: Relation to melanocarcinogenesis. *J Investig Dermatol* **117:** (6) 1471.

Van Ruissen F, Jansen BJH, De Jongh GJ, Van Vlijmen-Willems IMJJ, Schalkwijk J. 2001. Univ Med Ctr St Radboud, Dept Dermatol, POB 9101, NL-6500 HB Nijmegen, The Netherlands. Differential gene expression in premalignant human epidermis revealed by cluster analysis of serial analysis of gene expression (SAGE) libraries. *FASEB J* **16:** (2) 246.

Van't Veer LJ, Dai H, Van de Vijver MJ, He YD, Hart AA, Mao M, Peterse HL, Van der Kooy K, Marton MJ, Witteveen AT *et al*. 2002. c/o Friend SH, Rosetta Inpharmatics, 12040 115th Ave NE, Kirkland, Wa 98034, USA. Gene expression profiling predicts clinical outcome of breast cancer. *Nature* **415:** (6871) 530.

Wang HM, Wang H, Zhang W, Fuller GN*. 2002. *Univ Texas, MD Anderson Canc Ctr, Dept Pathol 085, 1515 Holcombe Blvd, Houston, Tx 77030, USA. Tissue microarrays: Applications in neuropathology research, diagnosis, and education. *Brain Pathol* **12:** (1) 95.

Weinstein JN. 2001. NIH, Bldg 37, Rm 4E-28, 9000 Rockville Pike, Bethesda, Md 20892, USA. Searching for pharmacogenomic markers: The synergy between omic and hypothesis-driven research. *Dis Marker* **17:** (2) 77.

Worsham MJ, Pals G, Raju U, Wolman SR. 2002. Henry Ford Hosp, Dept Otolaryngol, 2799 West Grand Blvd, Detroit, Mi 48202, USA. Establishing a molecular continuum in breast cancer: DNA microarrays and benign breast disease. *Cytometry* **47:** (1) 56.

Xerri L, Dales JP, Devilard E, Hassoun J, Birg F. 2001. Inst J Paoli-Calmettes, Dept Pathol, 232 Blvd St Marguerite, BP 156, FR-13273 Marseille 9, France. "Apoptosis" macro-array analysis of gene expression profiling in lymph node lesions (French, English Abstract). *Bull Acad Natl Med* **185:** (5) 963.

Xu XR, Huang J, Xu ZG, Qian BZ, Zhu ZD, Yan Q, Cai T, Zhang X, Xiao HS, Qu J *et al*. 2001. c/o Han ZG, Chinese Natl Human Genome Ctr, 351 Guo Shou Jing Rd, CN-201203 Shanghai, Peoples Rep China. Insight into hepatocellular carcinogenesis at transcriptome level by comparing gene expression profiles of hepatocellular carcinoma with those of corresponding noncancerous liver. *Proc Natl Acad Sci U S A* **98:** (26) 15089.

Yanagawa R, Furukawa Y, Tsunoda T, Kitahara O, Kameyama M, Murata K, Ishikawa O, Nakamura Y*. 2001. *Univ Tokyo, Inst Med Sci, Ctr Human Genome, Mol Med Lab, Minato ku, 4-6-1 Shirokanedai, Tokyo 108 8639, Japan. Genome-wide screening of genes showing altered expression in liver metastases of human colorectal cancers by cDNA microarray. *Neoplasia* 3: (5) 395.

Copyright © 2002 John Wiley & Sons, Ltd. 

### 8 EST, cDNA and other clone resources

Hukriede N, Fisher D, Epstein J, Joly L, Tellis P, Zhou Y, Barbazuk B, Cox K, Fenton-Noriega L, Hersey C *et al.* 2001. c/o Ekker M, Ottawa Civic Hosp, Res Inst, Ottawa, Ontario, Canada K1Y 4E9. The LN54 radiation hybrid map of zebrafish expressed sequences. *Genome Res* **11:** (12) 2127.

Mu XQ, Zhao S, Pershad R, Hsieh TF, Scarpa A, Wang SW, White RA, Beremand PD, Thomas TL, Gan L, Klein WH*. 2001. *Univ Texas, MD Anderson Canc Ctr, Dept Biochem & Mol Biol, Box 117, 1515 Holcombe Blvd, Houston, Tx 77030, USA. Gene expression in the developing mouse retina by EST sequencing and microarray analysis. *Nucleic Acids Res* **29:** (24) 4983.

Shelton D, Leach D, Baverstock P, Henry R. 2002. Sthn Cross Univ, Ctr Plant Conservat Genet, Lismore, NSW 2480, Australia. Isolation of genes involved in secondary metabolism from *Melaleuca alternifolia* (Cheel) using expressed sequence tags (ESTs). *Plant Sci* **162:** (1) 9.

Takasuga A, Hirotsune S, Itoh R, Jitohzono A, Suzuki H, Aso H, Sugimoto Y*. 2001. *Shirakawa Inst Anim Genet, Fukushima 961 8061, Japan. Establishment of a high throughput EST sequencing system using poly(A) tail-removed, cDNA libraries and determination of 36,000 bovine ESTs (Online Paper). *Nucleic Acids Res* **29:** (22) E108.

Terrier N, Ageorges A, Abbal P, Romieu C. 2001. UMR Sci Oenol II, Inst Natl Rech Agronom, 2 Pl Viala, FR-34060 Montpellier 01, France. Generation of ESTs from grape berry at various developmental stages. *J Plant Physiol* **158:** (12) 1575.

### 9 Functional genomics

Akerley BJ, Rubin EJ, Novick VL, Amaya K, Judson N, Mekalanos JJ*. 2002. *Harvard Univ, Sch Med, Dept Microbiol & Mol Genet, Bldg D1, Room 411, 200 Longwood Ave, Boston, Ma 02115, USA. A genome-scale analysis for identification of genes required for growth or survival of *Haemophilus influenzae*. *Proc Natl Acad Sci U S A* **99:** (2) 966.

Bianchi MM, Ngo S, Vandenbol M, Sarton G, Morlupi A, Ricci C, Stefani S, Marlino GB, Hilger F, Carignani G, Slonimski PP, Frontali L. 2001. Univ Rome La Sapienza, Dept Cell & Dev Biol, Ple Aldo Moro 5, IT-00185 Rome, Italy. Large-scale phenotypic analysis reveals identical contributions to cell functions of known and unknown yeast genes. *Yeast* **18:** (15) 1397.

Boucher L, Ouzounis CA, Enright AJ, Blencowe BJ*. 2001. *Univ Toronto, Charles H Best Inst, Banting & Best Dept Med Res, 112 Coll St, Room 410, Toronto, Ontario, Canada M5G 1L6. A genome-wide survey of RS domain proteins. *RNA* **7:** (12) 1693.

Boulton SJ, Gartner A, Reboul J, Vaglio P, Dyson N, Hill DE, Vidal M*. 2002. *Dana Farber Canc Inst, Boston, Ma 02115, USA. Combined functional genomic maps of the *C. elegans* DNA damage response. *Science* **295:** (5552) 127.

Brahmachari SK. 2001. Ctr Biochem Technol, Funct Genomic Unit, Mall Rd, IN-110007 Delhi, India. Keynote: Functional genomics and DNA chips. *Appl Biochem Biotechnol* **96:** (1-3) 9.

Chang YL, Tao QZ, Scheuring C, Ding KJ, Meksem K, Zhang HB*. 2001. *Texas A&M Univ, Dept Soil & Crop Sci, 2123 Tamus, College Station, Tx 77843, USA. An integrated MAP of *Arabidopsis thaliana* for functional analysis of its genome sequence. *Genetics* **159:** (3) 1231.

Cravchik A, Subramanian G, Broder S, Venter JC. 2001. Celera Genomics, 45 West Gude Dr, Rockville, Md 20850, USA. Sequence analysis of the human genome: Implications for the understanding of nervous system function and disease. *Arch Neurol* **58:** (11) 1772.

Draper J, Mur LAJ, Jenkins G, Ghosh Biswas GC, Bablak P, Hasterok R, Routledge APM. 2001. Univ Wales, Inst Biol Sci, Edward Llwyd Bldg, Aberystwyth SY23 3DA, Wales. *Brachypodium distachyon*. A new model system for functional genomics in grasses. *Plant Physiol* **127:** (4) 1539.

Gavin AC, Bosche M, Krause R, Grandi P, Marzioch M, Bauer A, Schultz J, Rick JM, Michon AM, Cruciat CM *et al.* 2002. Cellzome AG, Meyerhofstr 1, DE-69117 Heidelberg, Germany. Functional organization of the yeast proteome by systematic analysis of protein complexes. *Nature* **415:** (6868) 141.

Hemby SE, Sanchez MM, Winslow JT. 2001. Emory Univ, Sch Med, Dept Pharmacol, Yerkes Reg Primate Res Ctr, 954 Gatewood Rd NE, Atlanta, Ga 30329, USA. Functional Genomics approaches to a primate model of autistic symptomology. *J Autism Dev Disord* **31:** (6) 551.

Hippler M, Klein J, Fink A, Allinger T, Hoerth P. 2001. Univ Jena, Inst Allgemeine Bot & Pflanzenphysiol, Dornburger Str 159, DE-07743 Jena, Germany. Towards functional proteomics of membrane protein complexes: Analysis of thylakoid membranes from *Chlamydomonas reinhardtii*. *Plant J* **28:** (5) 595.

Ho Y, Gruhler A, Heibut A, Bader GD, Moore L, Adams SL, Millar A, Taylor P, Bennett K, Boutilier K *et al.* 2002. c/o Figeys DMDS Proteomics, 251 Attwell Dr, Toronto, Ontario, Canada M9W 7H4. Systematic identification of protein complexes in *Saccharomyces cerevisiae* by mass spectrometry. *Nature* **415:** (6868) 180.

Honma K, Ochiya T, Nagahara S, Sano A, Yamamoto H, Hirai K, Aso Y, Terada M*. 2001. *Natl Canc Ctr, Res Inst, Chuo ku, 1-1 Tsukiji, 5 chome, Tokyo 104 0045, Japan. Atelocollagen-based gene transfer in cells allows high-throughput screening of gene functions. *Biochem Biophys Res Commun* **289:** (5) 1075.

Jansen R, Greenbaum D, Gerstein M*. 2002. *Yale Univ, Dept Mol Biophys & Biochem, POB 6666, New Haven, Ct 06520, USA. Relating whole-genome expression data with protein-protein interactions. *Genome Res* **12:** (1) 37.

Karlyshev AV, Oyston PCF, Williams K, Clark GC, Titball RW, Winzeler EA, Wren BW*. 2001. *Univ London, London Sch Hyg & Trop Med, Dept Infect & Trop Dis, Keppel St, London WC1E 7HT, England. Application of high-density array-based signature-tagged mutagenesis to discover novel *Yersinia* virulence-associated genes. *Infect Immun* **69:** (12) 7810.

Kim M, Jiang LH, Wilson HL, North RA*, Surprenant A. 2001. *Univ Sheffield, Inst Mol Physiol, Alfred Denny Bldg, Sheffield S10 2TN, England. Proteomic and functional evidence for a $P2X_7$ receptor signalling complex. *EMBO J* **20:** (22) 6347.

Kumar A, Harrison PM, Cheung KH, Lan N, Echols N, Bertone P, Miller P, Gerstein MB, Snyder M*. 2002. *Yale Univ, Dept Mol Cellular & Dev Biol, POB 208103, New Haven, Ct 06520, USA. An integrated approach for finding overlooked genes in yeast. *Nat Biotechnol* **20:** (1) 58.

Loftus SK, Larson DM, Watkins-Chow D, Church DM, Lavan WJ. 2001. NIH/NHGRI, Mouse Embryol Sect, Genet Dis Res Inst, Bldg 49, Room 4A67, 49 Convent Dr, MSC 4472, Bethesda, Md 20892, USA. Generation of RCAS vectors useful for functional genomic analyses. *DNA Res* **8:** (5) 221.

Markstein M, Markstein P, Markstein V, Levine MS*. 2002. *Univ Calif Berkeley, Dept Mol & Cell Biol, Div Genet & Dev, 401 Barker Hall, Berkeley, Ca 94720, USA. Genome-wide analysis of clustered dorsal binding sites identifies putative target genes in the *Drosophila* embryo. *Proc Natl Acad Sci U S A* **99:** (2) 763.

Mattei B, Cervone F, Roepstorff P. 2001. Univ Rome La Sapienza, Dipt Biol Veg, Piazzale Aldo Moro 5, IT- 00185 Rome, Italy. The interaction between endopolygalacturonase from *Fusarium moniliforme* and PGIP from *Phaseolus vulgaris* studied by surface plasmon resonance and mass spectrometry. *Comp Funct Genom* **2:** (6) 359.

Momose Y, Iwahashi H*. 2001. *Natl Inst Adv Ind Sci & Technol, Natl Inst Biosci & Human Technol, Human Stress Signal Res Ctr, Tsukuba, Ibaraki 305 8566, Japan. Bioassay of cadmium using a DNA microarray: Genome-wide expression patterns of *Saccharomyces cerevisiae* response to cadmium. *Environ Toxicol Chem* **20:** (10) 2353.

Nekrutenko A, Makova KD, Li WH*. 2002. *Univ Chicago, Dept Ecol & Evolut, Chicago, Il 60637, USA. The $K_A/K_S$ ratio test for assessing the protein-coding potential of genomic regions: An empirical and simulation study. *Genome Res* **12:** (1) 198.

Popovici C, Leveugle M, Birnbaum D*, Coulier F. 2001. *INSERM, Oncol Mol Biol, U119 IFR57, 27 Blvd Lei Roure, FR-13009 Marseille, France. Coparalogy: Physical and functional clusterings in the human genome. *Biochem Biophys Res Commun* **288:** (2) 362.

Quintero MJ, Montesinos ML, Herrero A, Flores E*. 2001. *Univ Sevilla, Inst Bioquim Vegetal & Fotosintesis, CSIC, ES-41092 Seville, Spain. Identification of genes encoding amino acid permeases by inactivation of selected ORFs from the *Synechocystis* genomic sequence. *Genome Res* **11:** (12) 2034.

Seta KA, Kim R, Kim HW, Millhorn DE*, Beitner-Johnson D. 2001. *Univ Cincinnati, Coll Med, Dept Cellular & Mol Physiol, POB 67-0576, Cincinnati, Oh 45267, USA. Hypoxia-induced regulation of MAPK phosphatase-1 as identified by subtractive suppression hybridization and cDNA microarray analysis. *J Biol Chem* **276:** (48) 44405.

Stoll M, Cowley AW, Tonellato PJ, Greene AS, Kaldunski ML, Roman

Copyright © 2002 John Wiley & Sons, Ltd. 

RJ, Dumas P, Schork NJ, Wang ZT, Jacob HJ*. 2001. *Med Coll Wisconsin, Dept Physiol, 8701 Watertown Plank Rd, Milwaukee, Wi 53226, USA. A genomic-systems biology map for cardiovascular function. *Science* **294:** (5547) 1723.

Tong AHY, Evangelista M, Parsons AB, Xu H, Bader GD, Page N, Robinson M, Raghibizadeh S, Hogue CWV, Bussey H, Andrews B*, Tyers M, Boone C. 2001. *Univ Toronto, Dept Med Genet & Microbiol, 100 Coll St, Toronto, Ontario, Canada M5S 1A8. Systematic genetic analysis with ordered arrays of yeast deletion mutants. *Science* **294:** (5550) 2364.

Tsujimoto G, Katsuma S, Shiojima S, Hirasawa A, Tanoue A. 2001. Natl Children's Med Res Ctr, Dept Mol Cell Pharmacol, Setagaya ku, 3-35-31 Taishi do, Tokyo 154, Japan. Functional genomic research of $\alpha_1$-adrenoceptors. *J Cardiovasc Pharmacol* **38:** (Suppl 1) S1.

Wu G, Gu Y, Li SD, Yang ZB*. 2001. *Univ Calif Riverside, Dept Bot & Plant Sci, Riverside, Ca 92521, USA. A genome-wide analysis of *Arabidopsis* Rop-interactive CRIB motif-containing proteins that act as Rop GTPase targets. *Plant Cell* **13:** (12) 2841.

### 10 Transcriptomics

Ackermann K, Waxmann A, Glover CVC, Pyerin W. 2001. Deutsch Krebsforschungszentrum, Neuenheimer Feld 280, DE-69120 Heidelberg, Germany. Genes targeted by protein kinase CK2: A genome-wide expression array analysis in yeast. *Mol Cell Biochem* **227:** (1-2) 59.

Blanchard RK, Moore JB, Green CL, Cousins RJ*. 2001. *Univ Florida, Dept Food Sci & Human Nutr, Gainesville, Fl 32611, USA. Modulation of intestinal gene expression by dietary zinc status: Effectiveness of cDNA arrays for expression profiling of a single nutrient deficiency. *Proc Natl Acad Sci U S A* **98:** (24) 13507.

Cai NS, McCoy MT, Cadet JL*. 2001. *NIH/NIDA Mol Neuropsychiat Sect, Intramural Res Program, 5500 Nathan Shock Dr, Baltimore, Md 21224, USA. Profile of genes showing increased expression following dopamine and methamphetamine exposure in an immortalized neuronal cell line. *Restor Neurol Neurosci* **18:** (2-3) 57.

Caldwell R, Sapolsky R, Weyler W, Maile RR, Causey SC, Ferrari E*. 2001. *Genencor Int Inc, 925 Page Mill Rd, Palo Alto, Ca 94304, USA. Correlation between *Bacillus subtilis* scoC phenotype and gene expression determined using microarrays for transcriptome analysis. *J Bacteriol* **183:** (24) 7329.

De Nobel H, Lawrie L, Brul S, Klis F, Davis M, Alloush H, Coote P*. 2001. *Univ St Andrews, Ctr Biomolec Sci, North Haugh, St Andrews KY16 9ST, Scotland. Parallel and comparative analysis of the proteome and transcriptome of sorbic acid-stressed *Saccharomyces cerevisiae*. *Yeast* **18:** (15) 1413.

Dunaeva M, Adamska W*. 2001. *Univ Stockholm, Arrhenius Labs Nat Sci, Dept Biochem & Biophys, SE-10691 Stockholm, Sweden. Identification of genes expressed in response to light stress in leaves of *Arabidopsis thaliana* using RNA differential display. *Eur J Biochem* **268:** (21) 5521.

Fukusaki E, Oishi T, Tanaka H, Kajiyama S, Kobayashi A. 2001. Osaka Univ, Grad Sch Engn, Dept Biotechnol, 2-1 Yamadaoka, Suita, Osaka 565 0871, Japan. Identification of genes induced by taxol application using a combination of differential display RT-PCR and DNA microarray analysis. *Z Naturforsch C* **56:** (9-10) 814.

Ge H, Liu ZH, Church GM, Vidal M*. 2001. *Harvard Univ, Sch Med, Dana Farber Canc Inst, Boston, Ma 02115, USA. Correlation between transcriptome and interactome mapping data from *Saccharomyces cerevisiae*. *Nat Genet* **29:** (4) 482.

Heidenreich B, Mayer K, Sandermann H, Ernst D*. 2001. *GSF Natl Res Ctr Environm & Hlth, Inst Biochem Plant Pathol, Ingolstadter Landstr 1, DE-85764 Neuherberg, Germany. Mercury-induced genes in *Arabidopsis thaliana*: Identification of induced genes upon long-term mercuric ion exposure. *Plant Cell Environ* **24:** (11) 1227.

Irwin LN. 2001. Univ Texas, Dept Biol Sci, El Paso, Tx 79968, USA. Gene expression in the hippocampus of behaviorally stimulated rats: Analysis by DNA microarray. *Mol Brain Res* **96:** (1-2) 163.

Lee JM, Zhang SH, Saha S, Anna SS, Jiang C, Perkins J*. 2001. *F Hoffmann La Roche & Co Ltd, Dept VFB, Bldg 203-20A, CH-4070 Basel, Switzerland. RNA expression analysis using an antisense *Bacillus subtilis* genome array. *J Bacteriol* **183:** (24) 7371.

Perez-Amador MA, Lidder P, Johnson MA, Landgraf J, Wisman E, Green PJ*. 2001. *Michigan State Univ, Dept Energy Plant Res Lab, East Lansing, Mi 48824, USA. New molecular phenotypes in the dst mutants of *Arabidopsis* revealed by DNA microarray analysis. *Plant Cell* **13:** (12) 2703.

Rao L, Puschner B*, Prolla TA. 2001. *Univ Wisconsin, Dept Genet, Madison, Wi 53706, USA. Gene expression profiling of low selenium status in the mouse intestine: Transcriptional activation of genes linked to DNA damage, cell cycle control and oxidative stress. *J Nutr* **131:** (12) 3175.

Thimm O, Essigmann B, Kloska S, Altmann T, Buckhout TJ*. 2001. *Humboldt Univ, Invalidenstr 42, DE-10115 Berlin, Germany. Response of *Arabidopsis* to iron deficiency stress as revealed by microarray analysis. *Plant Physiol* **127:** (3) 1030.

Weitzel JM, Radtke C, Seitz HJ. 2001. Univ Klinikum Hamburg Eppendorf, Inst Med, Biochem & Mol Biol, Martinistr 52, DE-20246 Hamburg, Germany. Two thyroid hormone-mediated gene expression patterns *in vivo* identified by cDNA expression arrays in rat. *Nucleic Acids Res* **29:** (24) 5148.

Yang L, Tran DK, Wang X. 2001. Syngenta Res & Technol, Torrey Mesa Res Inst, Dept RNA Dynam, San Diego, Ca 92121, USA. BADGE, BeadsArray for the Detection of Gene Expression, a high-throughput diagnostic bioassay. *Genome Res* **11:** (11) 1888.

### 11 Proteomics

Ardekani AM, Herman EH, Sistare FD, Liotta LA, Petricoin EF*. 2001. *NCI/US/FDA, Div Therapeut Prod, Clin Proteomics Program, Ctr Biol Evaluat & Res, 8800 Rockville Pike, Bethesda, Md 20892, USA. Molecular profiling of cancer and drug-induced toxicity using new proteomic technologies. *Curr Ther Res* **62:** (11) 803.

Berry DA, Keogh A, Dos Remedios CG. 2001. Univ Sydney, Dept Anat & Histol, Inst Biomed Res, Sydney, NSW 2006, Australia. Nuclear membrane proteins in failing human dilated cardiomyopathy. *Proteomics* **1:** (12) 1507.

Bestel-Corre G, Dumas-Gaudot E*, Poinsot V, Dieu M, Dierick JF, Van Tuinen D, Remacle J, Gianinazzi-Pearson V, Gianinazzi S. 2002. *UMR INRA Univ Bourgogne, BBCEIPM, CMSE-INRA, BP 86510, FR-21065 Dijon, France. Proteome analysis and identification of symbiosis-related proteins from *Medicago truncatula* Gaertn by two-dimensional electrophoresis and mass spectrometry. *Electrophoresis* **23:** (1) 122.

Blank PS, Sjomeling CM, Backlund PS, Yergey AL*. 2002. *NIH/NICHHD, Lab Cellular & Mol Biophys, Bldg 10, Room 9D52, MSC 1580, Bethesda, Md 20892, USA. Use of cumulative distribution functions to characterize mass spectra of intact proteins. *J Am Soc Mass Spectrom* **13:** (1) 40.

Colello RJ, Fuss B, Fox MA, Alberti J. 2002. Virginia Commonwealth Univ, Med Coll Virginia, Dept Anat, POB 980709, Richmond, Va 23298, USA. A proteomic approach to rapidly elucidate oligodendrocyte-associated proteins expressed in the myelinating rat optic nerve. *Electrophoresis* **23:** (1) 144.

Coonrod SA, Wright PW, Herr JC. 2002. Univ Virginia, Dept Cell Biol, Box 439, Charlottesville, Va 22908, USA. Oolemmal proteomics. *J Reprod Immunol* **53:** (1-2) 55.

Escobar-Henriques M, Balguerie A, Monribot C, Boucherie H, Daignan-Fornier B*. 2001. *CNRS UMR 5095, Inst Biochem & Genet Cellulaires, 1 rue Camille St Saens, FR-33077 Bordeaux, France. Proteome analysis and morphological studies reveal multiple effects of the immunosuppressive drug mycophenolic acid specifically resulting from guanylic nucleotide depletion. *J Biol Chem* **276:** (49) 46237.

Georgiou HM, Rice GE, Baker MS. 2001. Mercy Hosp Women, Dept Obstet & Gynaecol, 126 Clarendon St, Melbourne, Vic 3002, Australia. Proteomic analysis of human plasma: Failure of centrifugal ultrafiltration to remove albumin and other high molecular weight proteins. *Proteomics* **1:** (12) 1503.

Ghafouri B, Stahlbom B, Tagesson C, Lindahl M*. 2002. *Linkoping Univ, Fac Hlth Sci, Div Environm & Occupat Med, SE-58185 Linkoping, Sweden. Newly identified proteins in human nasal lavage fluid from non-smokers and smokers using two-dimensional gel electrophoresis and peptide mass fingerprinting. *Proteomics* **2:** (1) 112.

Grant SGN, Blackstock WP. 2001. Univ Edinburgh, Dept Neurosci, 1 George Sq, Edinburgh EH8 9JZ, Scotland. Proteomics in neuroscience: From protein to network. *J Neurosci* **21:** (21) 8315.

Grus FH, Augustin AJ, Pfeiffer N, Schmidt-Erfurth U. 2001. Univ Mainz, Augenklin, Langenbeckstr 1, DE-55101 Mainz, Germany.

Copyright © 2002 John Wiley & Sons, Ltd.

From genome to proteome (German, English Abstract). *Ophthalmologe* **98:** (12) 1132.

Guillaume E, Evrard B, Com E, Moertz E, Jegou B, Pineau C*. 2001. *Univ Rennes 1, GERM-INSERM U435, Campus Beaulieu, FR-35042 Rennes, France. Proteome analysis of rat spermatogonia: Reinvestigation of stathmin spatio-temporal expression within the testis. *Mol Reprod Dev* **60:** (4) 439.

Hanash S, Brichory F, Beer D. 2001. Univ Michigan, Med Ctr, Dept Pediat, 1150 West Med Ctr Dr, Med Sci Res Bldg 1, Ann Arbor, Mi 48109, USA. A proteomic approach to the identification of lung cancer markers. *Dis Marker* **17:** (4) 295.

Holden JF, Poole FL, Tollaksen SL, Giometti CS, Lim H, Yates JR, Adams MWW*. 2001. *Univ Georgia, Dept Biochem & Mol Biol, Life Sci Bldg, Athens, Ga 30602, USA. Identification of membrane proteins in the hyperthermophilic archaeon *Pyrococcus furiosus* using proteomics and prediction programs. *Comp Funct Genom* **2:** (5) 275.

Huber CG, Timperio AM, Zolla L. 2001. Leopold Franzens Univ, Inst Analyt Chem & Radiochem, Innrain 52A, AU-6020 Innsbruck, Austria. Isoforms of photosystem II antenna proteins in different plant species revealed by liquid chromatography-electrospray ionization mass spectrometry. *J Biol Chem* **276:** (49) 45755.

Jacobs DI, Van Rijssen MS, Van der Heijden R, Verpoorte R. 2001. Gorlaeus Labs, Leiden Amsterdam Ctr Drug Res, Div Pharmacognosy, POB 9502, NL-2300 RA Leiden, The Netherlands. Sequential solubilization of proteins precipitated with trichloroacetic acid in acetone from cultured *Catharanthus roseus* cells yields 52% more spots after two-dimensional electrophoresis. *Proteomics* **1:** (11) 1345.

Jessen F, Lametsch R, Bendixen E, Kjaersgard IVH, Jorgensen BM. 2002. Danish Inst Fisheries & Marine Res, Dept Seafood Res, DTU Bldg 221, DK-2800 Lyngby, Denmark. Extracting information from two-dimensional electrophoresis gels by partial least squares regression. *Proteomics* **2:** (1) 32.

Jones MB, Krutzsch H, Shu HJ, Zhao YM, Liotta LA, Kohn EC, Petricoin EF*. 2002. *Bldg 29A, Room 2B02, 8800 Rockville Pike, Bethesda, Md 20892, USA. Proteomic analysis and identification of new biomarkers and therapeutic targets for invasive ovarian cancer. *Proteomics* **2:** (1) 76.

Junaid MA, Pullarkat RK*. 2001. *New York State Inst Basic Res Dev Disabil, Dept Dev Biochem, 1050 Forest Hill Rd, Staten Island, NY 10314, USA. Proteomic approach for the elucidation of biological defects in autism. *J Autism Dev Disord* **31:** (6) 557.

Kabir MM, Shimizu K*. 2001. *Kyushu Inst Technol, Dept Biochem Engn & Sci, Fukuoka 820 8502, Japan. Proteome analysis of a temperature-inducible recombinant *Escherichia coli* for poly-β-hydroxybutyrate production. *J Biosci Bioeng* **92:** (3) 277.

Knox S, Melrose J, Whitelock J. 2001. CSIRO, Div Mol Sci, 2 Richardson Pl, Riverside Corp Pk, Delhi Rd, Nth Ryde, NSW 2113, Australia. Electrophoretic, biosensor, and bioactivity analyses of perlecans of different cellular origins. *Proteomics* **1:** (12) 1534.

Kovarova H, Halada P, Man P, Golovliov I, Krocova Z, Spacek J, Porkertova S, Necasova R. 2002. Perkyne Milit Med Acad, Inst Radiobiol & Immunol, Trebesska 1575, CZ-50001 Hradec Kralove, Czech Republic. Proteome study of *Francisella tularensis* live vaccine strain-containing phagosome in Bcg/Nramp1 congenic macrophages: Resistant allele contributes to permissive environment and susceptibility to infection. *Proteomics* **2:** (1) 85.

Kruft V, Eubel H, Jansch L, Werhahn W, Braun HP*. 2001. *Univ Hannover, Inst Angew Genet, DE-30419 Hannover, Germany. Proteomic approach to identify novel mitochondrial proteins in *Arabidopsis*. *Plant Physiol* **127:** (4) 1694.

Lametsch R, Bendixen E*. 2001. *Danish Inst Agr Sci, Dept Anim Prod Qual, POB 50, DK-8830 Tjele, Denmark. Proteome analysis applied to meat science: Characterizing post mortem changes in porcine muscle. *J Agric Food Chem* **49:** (10) 4531.

Lampi KJ, Shih M, Ueda Y, Shearer TR, David LL*. 2002. *Oregon Hlth Sci Univ, Sch Dent, Dept Oral Mol Biol, 611 SW Campus Dr, Portland, Or 97201, USA. Lens proteomics: Analysis of rat crystallin sequences and two-dimensional electrophoresis map. *Invest Ophthalmol Vis Sci* **43:** (1) 216.

Le Naour F, Misek DE, Krause MC, Deneux L, Giordano TJ, Scholl S, Hanash SM*. 2001. *Univ Michigan, Sch Med, Dept Pediat, Box 0656, Ann Arbor, Mi 48109, USA. Proteomics-based identification of RS/DJ-1 as a novel circulating tumor antigen in breast cancer. *Clin Cancer Res* **7:** (11) 3328.

Liotta LA, Kohn EC, Petricoin EF. 2001. 10 Ctr Dr, MSC 1500, Bethesda, Md 20892, USA. Clinical proteomics: Personalized molecular medicine. *JAMA* **286:** (18) 2211.

Macdonald N, Chevalier S, Tonge R, Davison N, Rowlinson R, Young J, Rayner S, Roberts R*. 2001. *Syngenta Cent Toxicol Lab, Alderly Pk, Macclesfield SK10 4TJ, England. Quantitative proteomic analysis of mouse liver response to the peroxisome proliferator diethylhexylphthalate (DEHP). *Arch Toxicol* **75:** (7) 415.

Marques K, Sarazin B, Chane-Favre L, Zivy M, Thiellement H*. 2001. *Univ Paris Sud, INRA/CNRS/INA-PG, Stn Genet Vegetale, Gif-sur-Yvettes, France. Comparative proteomics to establish genetic relationships in the Brassicaceae family. *Proteomics* **1:** (11) 1457.

Martinovic S, Veenstra TD, Anderson GA, Pasa-Tolic L, Smith RD*. 2002. *Pacific NW Nat Labs, Environm & Molec Sci Lab, PO Box 999, Richland, Wa 99352, USA. Selective incorporation of isotopically labeled amino acids for identification of intact proteins on a proteome-wide level. *J Mass Spectrom* **37:** (1) 99.

Mathesius U, Keijzers G, Natera SHA, Weinman JJ, Djordjevic MA, Rolfe BG*. 2001. *Australian Natl Univ, Res Sch Biol Sci, Genomic Interact Grp, GPO Box 475, Canberra, ACT 2601, Australia. Establishment of a root proteome reference map for the model legume *Medicago truncatula* using the expressed sequence tag database for peptide mass fingerprinting. *Proteomics* **1:** (11) 1424.

McGregor E, Kempster L, Wait R, Welson SY, Gosling M, Dunn MJ, Powell JT. 2001. Proteome Sci plc, Inst Psychiat, KCL Sth Wing Lab, De Crespigny Pk, London SE5 8AF, England. Identification and mapping of human saphenous vein medial smooth muscle proteins by two-dimensional polyacrylamide gel electrophoresis. *Proteomics* **1:** (11) 1405.

Meehan KL, Holland JW, Dawkins HJS*. 2002. *Univ Western Australia, Royal Perth Hosp, Dept Surg, GPO Box X2213, Med Res Fdn Bldg, Perth, WA 6847, Australia. Proteomic analysis of normal and malignant prostate tissue to identify novel proteins lost in cancer. *Prostate* **50:** (1) 54.

Millar AH, Sweetlove LJ, Giege P, Leaver CJ. 2001. Univ Western Australia, Fac Med & Dent, Dept Biochem, Crawley, WA 6009, Australia. Analysis of the *Arabidopsis* mitochondrial proteome. *Plant Physiol* **127:** (4) 1711.

Montigiani S, Falugi F, Scarselli M, Finco O, Petracca R, Galli G, Mariani M, Manetti R, Agnusdei M, Cevenini R *et al*. 2002. c/o Grandi G, Chiron SpA, via Fiorentina 1, IT-53100 Siena, Italy. Genomic approach for analysis of surface proteins from *Chlamydia pneumoniae*. *Infect Immun* **70:** (1) 368.

Mortz E, Krogh TN, Vorum H, Gorg A. 2001. ACE BioSciences, Unsbjergvej 2A, DK-5220 Odense SO, Denmark. Improved silver staining protocols for high sensitivity protein identification using matrix-assisted laser desorption/ionization-time of flight analysis. *Proteomics* **1:** (11) 1359.

Nedelkov D, Nelson RW. 2001. Intrinsic Bioprobes Inc, 625 Sth Smith Rd, Suite 22, Tempe, Az 85281, USA. Delineation of *in vivo* assembled multiprotein complexes via biomolecular interaction analysis mass spectrometry. *Proteomics* **1:** (11) 1441.

Neverova I, Van Eyk JE*. 2002. *Queen's Univ, Dept Physiol, 429 Botterell Hall, Kingston, Ontario, Canada K7L 3N6. Application of reversed phase high performance liquid chromatography for subproteomic analysis of cardiac muscle. *Proteomics* **2:** (1) 22.

Norais N, Nogarotto R, Iacobini ET, Garaguso I, Grifantini R, Galli G, Grandi G*. 2001. *Chiron SpA, Dept Mol Biol, via Fiorentina 1, IT-53100 Siena, Italy. Combined automated PCR cloning, *in vitro* transcription/translation and two-dimensional electrophoresis for bacterial proteome analysis. *Proteomics* **1:** (11) 1378.

Nyman TA, Rosengren A, Syyrakki S, Pellinen TP, Rautajoki K, Lahesmaa R. 2001. Univ Turku, Ctr Biotechnol, POB 123, FI-20521 Turku, Finland. A proteome database of human primary T helper cells. *Electrophoresis* **22:** (20) 4375.

Predic J, Soskic V, Bradley D, Godovac-Zimmermann J*. 2002. *UCL, Dept Med, Ctr Mol Med, 5 Univ St, London WC1 6JJ, England. Monitoring of gene expression by functional proteomics: Response of human lung fibroblast cells to stimulation by endothelin-1. *Biochemistry* **41:** (3) 1070.

Randic M. 2001. 3225 Kingman Rd, Des Moines, Ia 50311, USA. On graphical and numerical characterization of proteomics maps. *J Chem Inform Comput Sci* **41:** (5) 1330.

Rodriguez JM, Salas ML, Santaren JF*. 2001. *Univ Autonoma Madrid, Fac Ciencias, Ctr Biol Mol Severo Ochoa, ES-28049 Madrid, Spain. African swine fever virus-induced polypeptides in porcine alveolar

Copyright © 2002 John Wiley & Sons, Ltd. 

macrophages and in Vero cells: Two-dimensional gel analysis. *Proteomics* **1:** (11) 1447.
Ruepp SU, Tonge RP, Shaw J, Wallis N, Pognan F*. 2002. *AstraZeneca, Mol & Invest Toxicol, 1800 Concord Pike, POB 15437, Wilmington, De 19850, USA. Genomics and proteomics analysis of acetaminophen toxicity in mouse liver. *Toxicol Sci* **65:** (1) 135.
Sandrock T, Poritz M, Kim M, Feldhaus MJ, Roth B, Caponigro G, Kamb A. 2002. De Hagen Proteomics Inc, Suite 300, 615 Arapeen Dr, Salt Lake City, Ut 84108, USA. Expression levels of transdominant peptides and proteins in *Saccharomyces cerevisiae*. *Yeast* **19:** (1) 1.
Schaffer S, Weil B, Nguyen VD, Dungmann G, Gunther K, Nickolaus M, Hermann T, Bott M*. 2001. *KFA Julich GmbH, Forschungszentrum, Inst Biotechnol 1, Angewandte Phys Chem Inst 7, Postfach 1913, DE-52425 Julich, Germany. A high-resolution reference map for cytoplasmic and membrane-associated proteins of *Corynebacterium glutamicum*. *Electrophoresis* **22:** (20) 4404.
Schaffer S, Isci N, Zickner B, Durre P*. 2002. *Univ Ulm, DE-89069 Ulm, Germany. Changes in protein synthesis and identification of proteins specifically induced during solventogenesis in *Clostridium acetobutylicum*. *Electrophoresis* **23:** (1) 110.
Seow TK, Korke R, Liang RCMY, Ong SE, Ou K, Wong K, Hu WS*, Chung MCM. 2001. *Univ Minnesota, Dept Chem Engn & Mat Sci, 421 Washington Ave SE, Minneapolis, Mn 55455, USA. Proteomic investigation of metabolic shift in mammalian cell culture. *Biotechnol Prog* **17:** (6) 1137.
Skylas DJ, Copeland L, Rathmell WG, Wrigley CW. 2001. Macquarie Univ, Australian Proteome Anal Facil, Nth Ryde, NSW 2109, Australia. The wheat-grain proteome as a basis for more efficient cultivar identification. *Proteomics* **1:** (12) 1542.
Soskic V, Godovac-Zimmermann J*. 2001. *UCL, Dept Med, Rayne Inst, Ctr Mol Med, 5 Univ St, London WC1 6JJ, England. Improvement of an in-gel tryptic digestion method for matrix-assisted laser desorption/ionization-time of flight mass spectrometry peptide mapping by use of volatile solubilizing agents. *Proteomics* **1:** (11) 1364.
Stewart II, Thomson T, Figeys D. 2001. MDS-Proteomics Inc, 251 Attwell Drive, Toronto, Ontario, Canada M9W 7H4. $^{18}O$ labeling: A tool for proteomics. *Rapid Commun Mass Spectrom* **15:** (24) 2456.
Thiel M, Bruchhaus I*. 2001. *Bernhard Nocht Inst Trop Med, Bernhard Nocht Str 74, DE-20359 Hamburg, Germany. Comparative proteome analysis of *Leishmania donovani* at different stages of transformation from promastigotes to amastigotes. *Med Microbiol Immunol* **190:** (1-2) 33.
Tilleman K, Stevens I, Spittaels K, Van den Haute C, Clerens S, Van den Bergh G, Geerts H, Val Leuven F, Vandesande F, Moens L*. 2002. *Univ Instelling Antwerp, Dept Biochem, Universiteitspl 1, BE-2610 Wilrijk, Belgium. Differential expression of brain proteins in glycogen synthase kinase-3β transgenic mice: A proteomics point of view. *Proteomics* **2:** (1) 94.
Ueda Y, Duncan MK, David LL*. 2002. *Oregon Hlth Sci Univ, Sch Dent, Dept Oral Mol Biol, 611 SW Campus Dr, Portland, Or 97201, USA. Lens proteomics: The accumulation of crystallin modifications in the mouse lens with age. *Invest Ophthalmol Vis Sci* **43:** (1) 205.
Utt M, Nilsson I, Ljungh A, Wadstrom T*. 2002. *Lund Univ, Dept Med Microbiol Dermatol & Infect, Solvegatan 23, SE-22362 Lund, Sweden. Identification of novel immunogenic proteins of *Helicobacter pylori* by proteome technology. *J Immunol Methods* **259:** (1-2) 1.
Vuadens F, Gasparini D, Deon C, Sanchez JC, Hochstrasser DF, Schneider P, Tissot JD*. 2002. *Serv Reg Vaudois Transfus Sanguine, Rue Bugnon 27, CH-1005 Lausanne, Switzerland. Identification of specific proteins in different lymphocyte populations by proteomic tools. *Proteomics* **2:** (1) 105.
Wandschneider S, Fehring V, Jacobs Emeis S, Thiesen HJ, Lohr M*. 2001. *Univ Heidelberg, Fak Klin Med Mannheim, Med Klin 2, Sekt Mol Gastroenterol, DE-68135 Mannheim, Germany. Autoimmune pancreatic disease: Preparation of pancreatic juice for proteome analysis. *Electrophoresis* **22:** (20) 4383.
Weiller GF, Djordjevic MJ, Caraux G, Chen HC, Weinman JJ. 2001. Australian Natl Univ, Res Sch Biol Sci, Genome Interact Grp, Canberra, ACT 0200, Australia. A specialised proteomic database for comparing matrix-assisted laser desorption/ionization-time of flight mass spectrometry data of tryptic peptides with corresponding sequence database segments. *Proteomics* **1:** (12) 1489.
Weng S, Gu K, Hammond PW, Lohse P, Rise C, Wagner RW, Wright MC, Kuimelis RG*. 2002. *Phylos, 128 Spring St, Lexington, Ma 02421, USA. Generating addressable protein microarrays with PROfusion™ covalent mRNA-protein fusion technology. *Proteomics* **2:** (1) 48.
West KA, Yan L, Miyagi M, Crabb JS, Marmorstein AD, Marmorstein L, Crabb JW*. 2001. *Cleveland Clin Fdn, Cole Eye Inst, Lerner Res Inst, 9500 Euclid Ave, Cleveland, Oh 44195, USA. Proteome survey of proliferating and differentiating rat RPE-J cells. *Exp Eye Res* **73:** (4) 479.
Wolters DA, Washburn MP, Yates JR*. 2001. *Torrey Mesa Res Inst, 3115 Merryfield Row, San Diego, Ca 92121, USA. An automated multidimensional protein identification technology for shotgun proteomics. *Anal Chem* **73:** (23) 5683.
Woodward AM, Kaderbhai N, Kaderbhai M, Shaw A, Rowland J, Kell DB*. 2001. *Univ Wales, Inst Biol Sci, Aberystwyth SY23 3DD, Wales. Histometrics: Improvement of the dynamic range of fluorescently stained proteins resolved in electrophoretic gels using hyperspectral imaging. *Proteomics* **1:** (11) 1351.
Zuo X, Speicher DW*. 2002. *Wistar Inst Anat & Biol, 3601 Spruce St, Philadelphia, Pa 19104, USA. Comprehensive analysis of complex proteomes using microscale solution isoelectrofocusing prior to narrow pH range two-dimensional electrophoresis. *Proteomics* **2:** (1) 58.

### 12 Protein structural genomics

Bhavesh NS, Panchal SC, Hosur RV*. 2001. *Tata Inst Fundamental Res, Dept Chem Sci, Homi Bhabha Rd, IN-400005 Mumbai, India. An efficient high-throughput resonance assignment procedure for structural genomics and protein folding research by NMR. *Biochemistry* **40:** (49) 14727.
Fogolari F, Tessari S, Molinari H. 2002. Univ Verona, Fac Sci MMFFNN, Dipt Sci Tecnol, Ca Vignal 1, Strada le Grazie 15, IT-37134 Verona, Italy. Singular value decomposition analysis of protein sequence alignment score data. *Protein-Struct Funct Genet* **46:** (2) 161.
Kuhn P, Soltis SM. 2001. Stanford Univ, POB 4349, MS69, Palo Alto, Ca 94304, USA. Macromolecular structure determination in the post-genome era. *Nucl Instrum Methods Phys Res A* **467:** (2) 1363.
Sivaraman J, Li YG, Plamondon J, Larocque R, Raymond S, Sauve V, Smith C, Boju L, Schrag J, Matte A *et al*. 2001. c/o Cygler M, Natl Res Council Canada, Biotechnol Res Inst, 6100 Royalmount Ave, Montreal, Quebec, Canada H4P 2R2. A structural genomics pilot project based on gene targets selected from *Escherichia coli*. *J Cryst Growth* **232:** (1-4) 421.

### 13 Metabolomics

Radivoyevitch T. 2001. Case Western Res Univ, Dept Epidemiol & Biostat, BRB G-19, Cleveland, Oh 44106, USA. Sphingoid base metabolism in yeast: Mapping gene expression patterns into qualitative metabolite time course predictions. *Comp Funct Genom* **2:** (5) 289.
Razeghi P, Young ME, Alcorn JL, Moravec CS, Frazier OH, Taegtmeyer H*. 2001. *Univ Texas, Sch Med, Dept Internal Med, Div Cardiol, 6431 Fannin, Houston, Tx 77030, USA. Metabolic gene expression in fetal and failing human heart. *Circulation* **104:** (24) 2923.

### 14 Genomic approaches to development

Chang B, Somogyi R, Fuhrman S*. 2001. *Molecular Mining Corp, 128 Ontario St, Kingston, Ontario, Canada K7L 2Y4. Evidence for shared genetic programs from cluster analysis of hippocampal gene expression dynamics in development and response to injury. *Restor Neurol Neurosci* **18:** (2-3) 115.
Hemberger M, Cross JC, Ropers HH, Lehracht H, Fundele R, Himmelbauer H. 2001. Univ Calgary, Dept Biochem & Mol Biol, 3330 Hosp Dr NW, Calgary, Alberta, Canada T2N 4N1. UniGene cDNA array-based monitoring of transcriptone changes during placental development mouse. *Proc Natl Acad Sci U S A* **98:** (23) 13126.
Kudoh T, Tsang M, Hukriede NA, Chen XF, Dedekian M, Clarke CJ, Kiang A, Schultz S, Epstein JA, Toyama R, David IB. 2001. *NIH/NICHHD, Mol Genet Lab, Bethesda, Md 20892, USA. A gene expression screen in zebrafish embryogenesis. *Genome Res* **11:** (12) 1979.
Loring JF, Porter JG, Seilhamer J, Kaser MR, Wesselschmidt R. 2001. Incyte Genomics, Dept Life Sci, 3160 Porter Dr, Palo Alto, Ca 94304,

Copyright © 2002 John Wiley & Sons, Ltd. 

USA. A gene expression profile of embryonic stem cells and embryonic stem cell-derived neurons. *Restor Neurol Neurosci* **18:** (2-3) 81.

Ma LG, Li JM, Qu LJ, Hager J, Chen ZL, Zhao HY, Deng XW*. 2001. *Peking Univ, Coll Life Sci, Peking Yale Joint Ctr Plant Mol Genetics & Agrobiotechnol, CN-100871 Beijing, Peoples Rep China. Light control of *Arabidopsis* development entails coordinated regulation of genome expression and cellular pathways. *Plant Cell* **13:** (12) 2589.

Shibata M, Itoh M, Ohmori S, Shinga J, Taira M*. 2001. *Univ Tokyo, Grad Sch Sci, Dept Biol Sci, Bunkyo ku, 7-3-1 Hongo, Tokyo 113 0033, Japan. Systematic screening and expression analysis of the head organizer genes in *Xenopus* embryos. *Dev Biol* **239:** (2) 241.

Theilhaber J, Connolly T, Roman-Roman S, Bushnell S, Jackson A, Call K, Garcia T, Baron R. 2002. Aventis Pharmaceut, Cambridge Genomics Ctr, Cambridge, Ma 02139, USA. Finding genes in the C2C12 osteogenic pathway by k-nearest-neighbor classification of expression data. *Genome Res* **12:** (1) 165.

Truckenmiller ME, Vawter MP, Cheadle C, Coggiano M, Donovan DM, Freed WJ, Becker KG. 2001. NIH/NIDA, Cellular Neurobiol, Res Branch, IRP, 5500 Nathan Shock Dr, Baltimore, Md 21224, USA. Gene expression profile in early stage of retinoic acid-induced differentiation of human SH-SY5Y neuroblastoma cells. *Restor Neurol Neurosci* **18:** (2-3) 67.

Zhou FC, Duguid JR, Edenberg HJ, McClintick J, Young P, Nelson P. 2001. Indiana Univ, Sch Med, Dept Anat & Cell Biol, MS 508, 635 Barnhill Dr, Indianapolis, In 46202, USA. DNA microarray analysis of differential gene expression of 6-year-old rat neural striatal progenitor cells during early differentiation. *Restor Neurol Neurosci* **18:** (2-3) 95.

### 15 Technological advances

Baba Y. 2001. Address not available. Genome polymorphism analysis and proteome analysis by microchip electrophoresis. *Electrochemistry* **69:** (9) 706.

Barrett T, Cheadle C, Wood WH, Teichberg D, Donovan DM, Freed WJ, Becker KG, Vawter MP. 2001. NIH/NIA, DNA Array Unit, Res Resources Branch, Rm 4-D16, 5600 Nathan Shock Dr, Baltimore, Md 21224, USA. Assembly and use of a broadly applicable neural cDNA microarray. *Restor Neurol Neurosci* **18:** (2-3) 127.

Beck MT, Holle L, Chen WY*. 2001. *Clemson Univ, Clemson, SC 29634, USA. Combination of PCR subtraction and cDNA microarray of differential gene expression profiling. *Biotechniques* **31:** (4) 782.

Bennett KL, Stensballe A, Podtelejnikov AV, Moniatte M, Jensen ON. 2002. MDS Proteomics A/S, Staermosegaardsvej 6, DK-5230 Odense M, Denmark. Phosphopeptide detection and sequencing by matrix-assisted laser desorption/ionization quadrupole time-of-flight tandem mass spectrometry. *J Mass Spectrom* **37:** (2) 179.

Catimel B, Rothacker J, Nice E*. 2001. *Royal Melbourne Hosp, Ludwig Inst Canc Res, Melbourne Tumour Biol Branch, POB 2008, Parkville, Vic 3050, Australia. The use of biosensors for microaffinity purification: An integrated approach to proteomics. *J Biochem Biophys Methods* **49:** (1-3) 289.

Chun MS, Kang D, Kim Y*, Chung D. 2001. *Kyungnam Univ, Dept Chem, Masan, South Korea. Protein analysis with large volume sample stacking with an electrosmotic flow pump: A potential approach for proteomics. *Microchem J* **70:** (3) 247.

Dupre I, El Atifi M, Rostaing B, Chambaz EM, Benabid AL, Berger E. 2001. Univ Grenoble 1, CHR Grenoble, INSERM U318, Lab Neurosci Preclin, BP 217X, FR-38043 Grenoble 09, France. Microarray RNA quantification assay for large-scale nanosamples analysis. *Biotechniques* **31:** (4) 856.

Griffin TJ, Han DKM, Gygi SP, Rist B, Lee H, Aebersold R, Parker KC. 2001. Inst Syst Biol, 4225 Roosevelt Way NE, Suite 200, Seattle, Wa 98105, USA. Toward a high-throughput approach to quantitative proteomic analysis: Expression-dependent protein identification by mass spectrometry. *J Am Soc Mass Spectrom* **12:** (12) 1238.

Herwig R, Aanstad P, Clark M, Lehrach H. 2001. Max-Planck-Inst Mol Genet, Ihnestr 73, DE-14195 Berlin, Germany. Statistical evaluation of differential expression on cDNA nylon arrays with replicated experiments (Online paper). *Nucleic Acids Res* **29:** (23) E117.

Hille JM, Freed AL, Watzig H*. 2001. *Tech Univ Braunschweig, Inst Pharmazeut Chem, Beethovenstr 55, DE-38106 Braunschweig, Germany. Possibilities to improve automation, speed and precision of proteome analysis: A comparison of two-dimensional electrophoresis and alternatives. *Electrophoresis* **22:** (19) 4035.

Huber M, Losert D, Hiller R, Harwanegg C, Mueller MW, Schmidt WM*. 2001. *VBC Genomics Biosci Res GmbH, Rennweg 958-5, AU-1030 Vienna, Austria. Detection of single base alterations in genomic DNA by solid phase polymerase chain reaction on oligonucleotide microarrays. *Anal Biochem* **299:** (1) 24.

Hunter TC, Yang L, Zhu HN, Majidi V, Bradbury EM, Chen X*. 2001. *Los Alamos Natl Lab, BN-2, MS M888, Los Alamos, NM 87544, USA. Peptide mass mapping constrained with stable isotope-tagged peptides for identification of protein mixtures. *Anal Chem* **73:** (20) 4891.

King HC, Sinha AA*. 2001. *Cornell Univ, Weill Med Coll, Dept Dermatol, 510 East 70th St, New York, NY 10021, USA. Gene expression profile analysis by DNA microarrays: Promise and pitfalls. *JAMA* **286:** (18) 2280.

Knapp DR, Kim JS. 2001. Med Univ Sth Carolina, Dept Pharmacol, 173 Ashley Ave, Charleston, SC 29425, USA. Inexpensive cleanroom for microfabrication, microarray work, and other activities requiring protection from airborne contamination. *Biotechniques* **31:** (4) 842.

Mayer C, Stich N, Schalkhammer T, Bauer G. 2001. Tech Univ Delft, Inst Analyt Biotechnol, Julianalaan 67, NL-2628 BC Delft, The Netherlands. Slide-format proteomic biochips based on surface-enhanced nanocluster-resonance. *Fresenius J Anal Chem* **371:** (2) 238.

Moya TFM, Newbold CJ, Wallace RJ, Moyano FJ. 2002. Univ Almeria, Dept Biol Aplicada, Area Biol Anim, Cite II-B, Campus Univ La Canada, ES-04120 Almeria, Spain. Effects of high-molecular-mass substrates on protein migration during sodium dodecyl sulfate-polyacrylamide gel electrophoresis. *Electrophoresis* **23:** (1) 1.

Nallur G, Luo CH, Fang LH, Cooley S, Dave V, Lambert J, Kukanskis K, Kingsmore S, Lasken R, Schweitzer B. 2001. Molecular Staging Inc, 300 George St, New Haven, Ct 06511, USA. Signal amplification by rolling circle amplification on DNA microarrays (Online paper). *Nucleic Acids Res* **29:** (23) E118.

Nesatyy VJ, Dacanay A, Kelly JF, Ross NW. 2002. National Res Council Canada, Inst Marine Biosci, Halifax, Nova Scotia, Canada B3H 3Z1. Microwave-assisted protein staining: Mass spectrometry compatible methods for rapid protein visualisation. *Rapid Commun Mass Spectrom* **16:** (4) 272.

Pabon C, Modrusan Z, Ruvolo MV, Coleman IM, Daniel S, Yue H, Arnold LJ, Reynolds MA*. 2001. *Incyte Genomics, 3160 Porter Ave, Palo Alto, Ca 94304, USA. Optimized T7 amplification system for microarray analysis. *Biotechniques* **31:** (4) 874.

Paegel BM, Emrich CA, Weyemayer GJ, Scherer JR, Mathies RA*. 2002. *Univ Calif Berkeley, Dept Chem, Berkeley, Ca 94720, USA. High throughput DNA sequencing with a microfabricated 96-lane capillary array electrophoresis bioprocessor. *Proc Natl Acad Sci U S A* **99:** (2) 574.

Peters EC, Horn DM, Tully DC, Brock A. 2001. Novartis Res Fdn, Genomics Inst, 3115 Merryfield Row, San Diego, Ca 92121, USA. A novel multifunctional labeling reagent for enhanced protein characterization with mass spectrometry. *Rapid Commun Mass Spectrom* **15:** (24) 2387.

Ramstrom M, Palmblad M, Amirkhani A, Tsybin YO, Markides KE, Hakansson P, Bergquist J*. 2001. *Uppsala Univ, Dept Analyt Chem, POB 531, SE-75121 Uppsala, Sweden. Micro-capillary liquid chromatography-Fourier transform ion cyclotron resonance mass spectrometry: A powerful tool for peptide and protein identification. *Acta Biochim Pol* **48:** (4) 1101.

Rosenow C, Saxena RM, Durst M, Gingeras TR. 2001. Affymetrix Inc, 3380 Cent Expressway, Santa Clara, Ca 95051, USA. Prokaryotic RNA preparation methods useful for high density array analysis: Comparison of two approaches (Online Paper). *Nucleic Acids Res* **29:** (22) E112.

Ruuska SA, Ohlrogge JB. 2001. Michigan State Univ, East Lansing, Mi 48824, USA. Protocol for small-scale RNA isolation and transcriptional profiling of developing *Arabidopsis* seeds. *Biotechniques* **31:** (4) 752.

Schober MS, Min YN, Chen YQ*. 2001. *Wayne State Univ, Dept Pathol, 540 East Canfield, Detroit, Mi 48201, USA. Serial analysis of gene expression in a single cell. *Biotechniques* **31:** (6) 1240.

Soldatov AV, Nabirochkina N, Georgieva SG, Eickhoff H*. 2001. *Scienion AG, Volmerstr 7A, DE-12989 Berlin, Germany. Adjustment of transfer tools for the production of micro- and macroarrays. *Biotechniques* **31:** (4) 848.

Toonen RJ, Hughes S. 2001. Univ Calif, Sect Evolut & Ecol, Davis, Ca 95616, USA. Increased throughput for fragment analysis on an ABI

Copyright © 2002 John Wiley & Sons, Ltd. 

PRISM 377 automated sequencer using a membrane comb and STRand software. *Biotechniques* **31:** (6) 1320.

Van Dam RM, Quake SR*. 2002. *Calif Inst Technol, Dept Appl Phys, Pasadena, Ca 91125, USA. Gene expression analysis with universal n-mer arrays. *Genome Res* **12:** (1) 145.

Walhagen K, Unger KK, Hearn MTW*. 2001. *Monash Univ, Ctr Bioproc Technol, Dept Biochem & Mol Biol, Clayton, Vic 3168, Australia. Capillary electrochromatography analysis of hormonal cyclic and linear peptides. *Anal Chem* **73:** (20) 4924.

Wu RA, Zou HF*, Ye ML, Lei ZD, Ni JY. 2001. *Acad Sinica, Dalian Inst Chem Phys, Natl Chromatog R&A Ctr, CN-116011 Dalian, Peoples Rep China. Capillary electrochromatography for separation of peptides driven with electrophoretic mobility on monolithic column. *Anal Chem* **73:** (20) 4918.

Zou W, Ueda M, Yamanaka H, Tanaka A*. 2001. *Kyoto Univ, Grad Sch Engn, Dept Synthet Chem & Biol Chem, Lab Appl Biol Chem, Sakyo ku, Kyoto 606 8501, Japan. Construction of a combinatorial protein library displayed on yeast cell surface using DNA random priming method. *J Biosci Bioeng* **92:** (4) 393.

### 16 Bioinformatics

Adiga PSU, Bhomra A, Turri MG, Nicod A, Datta SR, Jeavons P, Mott R, Flint J*. 2001. *Univ Oxford, Wellcome Trust Ctr Human Genet, Psychiat Genet Grp, Roosevelt Dr, Oxford OX3 7BN, England. Automatic analysis of agarose gel images. *Bioinformatics* **17:** (11) 1084.

Afonnikov DA, Oshchepkov DY, Kolchanov NA. 2001. Inst Cytol & Genet, Lab Theoret Genet, 10 Lavrentyev Ave, RU-630090 Novosibirsk, Russia. Detection of conserved physico-chemical characteristics of proteins by analyzing clusters of positions with co-ordinated substitutions. *Bioinformatics* **17:** (11) 1035.

Baggerly KA, Coombes KR, Hess KR, Stivers DN, Abruzzo LV, Zhang W. 2001. Univ Texas, MD Anderson Canc Ctr, Dept Biostat, 1515 Holcombe Blvd, Box 447, Houston, Tx 77030, USA. Identifying differentially expressed genes in cDNA microarray experiments. *J Comput Biol* **8:** (6) 639.

Batzoglou S, Jaffe DB, Stanley K, Butler J, Gnerre S, Mauceli E, Berger B, Mesirov JP, Lander ES*. 2002. *MIT, Whitehead Inst Biomed Res, Ctr Genome Res, Cambridge, Ma 02141, USA. ARACHNE: A whole-genome shotgun assembler. *Genome Res* **12:** (1) 177.

Bejerano G, Seldin Y, Margalit H, Tishby N. 2001. Hebrew Univ Jerusalem, Sch Engn & Comp Sci, IL-91904 Jerusalem, Israel. Markovian domain fingerprinting: Statistical segmentation of protein sequences. *Bioinformatics* **17:** (10) 927.

Bohr HG, Rogen P, Jalkanen KJ. 2001. Tech Univ Denmark, Dept Phys, DK-2800 Lyngby, Denmark. Applications of neural network prediction of conformational states for small peptides from spectra and of fold classes. *Comput Chem* **26:** (1) 65.

Bolten E, Schliep A*, Schneckener S, Schomburg D, Schrader R. 2001. *Univ Cologne, ZPR/ZAIK, Weyertal 80, DE-50937 Cologne, Germany. Clustering protein sequences-structure prediction by transitive homology. *Bioinformatics* **17:** (10) 935.

Brazma A, Hingamp P, Quackenbush J, Sherlock G, Spellman P, Stoeckert C, Aach J, Ansorge W, Ball CA, Causton HC *et al*. 2001. European Bioinformat Inst, EMBL Outstn, Wellcome Trust Genome Campus, Cambridge CB10 1SD, England. Minimum information about a microarray experiment (MIAME): Toward standards for microarray data. *Nat Genet* **29:** (4) 365.

Chan MS, Maiden MCJ, Spratt BG. 2001. Univ Oxford, John Radcliffe Hosp, Inst Mol Med, Dept Paediat, Oxford OX3 9DS, England. Database-driven multi locus sequence typing (MLST) of bacterial pathogens. *Bioinformatics* **17:** (11) 1077.

Dandekar T, Du FL, Schirmer RH, Schmidt S. 2001. European Mol Biol Lab, Meyerhofstr 1, POB 10.22.09, DE-69012 Heidelberg, Germany. Medical target prediction from genome sequence: Combining different sequence analysis algorithms with expert knowledge and input from artificial intelligence approaches. *Comput Chem* **26:** (1) 15.

Del Rio G, Bartley TF, Del Rio H, Rao R, Jin K, Greenberg DA, Eshoo M, Bredesen DE*. 2001. *Buck Inst Age Res, 8001 Redwood Blvd, Novato, Ca 94945, USA. Mining DNA microarray data using a novel approach based on graph theory. *FEBS Lett* **509:** (2) 230.

Douabin-Gicquel V, Soriano N, Ferran H, Wojcik F, Palierne E, Tamim S, Jovelin T, McKie AT, Le Gall JY, David V, Mosser J*. 2001. *Fac Med, Dept Biochim & Biol Mol, 2 Ave Pr Leon Bernard, CS 34317, FR-35043 Rennes, France. Identification of 96 single nucleotide polymorphisms in eight genes involved in iron metabolism: Efficiency of bioinformatic extraction compared with a systematic sequencing approach. *Hum Genet* **109:** (4) 393.

Far RKK, Nedbal W, Sczakiel G. 2001. Med Univ Lubeck, Inst Mol Med, Ratzeburger Allee 160, DE-23538 Lubeck, Germany. Concepts to automate the theoretical design of effective antisense oligonucleotides. *Bioinformatics* **17:** (11) 1058.

Fariselli P, Casadio R. 2001. Univ Bologna, Dept Biol, Lab Biophys, CIRB Biocomp Unit, via Irnerio 42, IT-40126 Bologna, Italy. Prediction of disulfide connectivity in proteins. *Bioinformatics* **17:** (10) 957.

Field HI, Fenyo D, Beavis RC. 2002. Proteometrics, POB 32323, London SW17 8JZ, England. RADARS, a bioinformatics solution that automates proteome mass spectral analysis, optimises protein identification, and archives data in a relational database. *Proteomics* **2:** (1) 36.

Frith MC, Hansen U, Weng ZP*. 2001. *Boston Univ, Bioinformatics Program, 44 Cummington St, Boston, Ma 02215, USA. Detection of *cis*-element clusters in higher eukaryotic DNA. *Bioinformatics* **17:** (10) 878.

Gilbert D, Westhead D, Viksna J, Thornton J. 2001. City Univ London, Dept Comp, London EC1V 0HB, England. A computer system to perform structure comparison using TOPS representations of protein structure. *Comput Chem* **26:** (1) 23.

Hatzigeorgiou AG, Fiziev P, Reczko M. 2001. Metagen GmbH, Ihnestr 63, DE-14195 Berlin, Germany. DIANA-EST: A statistical analysis. *Bioinformatics* **17:** (10) 913.

Ivanov V, Beniaminov A, Mikheyev A, Minyat E. 2001. Univ Alabama, Ctr Biophys Sci & Engn, 1530 3rd Ave Sth, Birmingham, Al 35294, USA. A mechanism for stop codon recognition by the ribosome: A bioinformatic approach. *RNA* **7:** (12) 1683.

Jia L, Ho NC, Park SS, Powell J, Francomano CA. 2001. MGB/NHGRI/NIH, 10/10C101, 9000 Rockville Pike, Bethesda, Md 20892, USA. Comprehensive resource: Skeletal gene database. *Am J Med Genet* **106:** (4) 275.

Jordan IK, Bishop GR*, Gonzalez DS. 2001. *Millsaps Coll, Dept Chem, Jackson, Ms 39210, USA. Sequence and structural aspects of functional diversification in class I α-mannosidase evolution. *Bioinformatics* **17:** (10) 965.

Kane MD. 2002. Genomics Solution Inc, 4355 Varsity Dr, Suite E, Ann Arbor, Mi 48108, USA. Aligning experimental design with bioinformatics analysis to meet discovery research objectives. *Cytometry* **47:** (1) 50.

Kielbasa SM, Korbel JO, Beule D, Schuchhardt J, Herzel H. 2001. Humboldt Univ, Invalidenstr 43, DE-10115 Berlin, Germany. Combining frequency and positional information to predict transcription factor binding sites. *Bioinformatics* **17:** (11) 1019.

Kraemer E, Wang J, Guo JH, Hopkins S, Arnold J. 2001. Univ Georgia, Dept Comp Sci, Athens, Ga 30602, USA. An analysis of gene-finding programs for *Neurospora crassa*. *Bioinformatics* **17:** (10) 901.

Kretschmann E, Fleischmann W, Apweiler R. 2001. European Bioinformatics Inst, EMBL Outstn, Wellcome Trust Genome Campus, Hinxton, Cambridge CB10 1SD, England. Automatic rule generation for protein annotation with the C4.5 data mining algorithm applied on SWISS-PROT. *Bioinformatics* **17:** (10) 920.

Kurtz S, Choudhuri JV, Ohlebusch E, Schleiermacher C, Stoye J, Giegerich R. 2001. Univ Bielefeld, Fac Technol, POB 10.01.31, DE-33501 Bielefeld, Germany. REPuter: The manifold applications of repeat analysis on a genomic scale. *Nucleic Acids Res* **29:** (22) 4633.

Lenhard B, Hayes WS, Wasserman WW*. 2001. *Karolinska Inst, Ctr Genomics & Bioinformatics, Stockholm, Sweden. GeneLynx: A gene-centric portal to the human genome. *Genome Res* **11:** (12) 2151.

Levitsky VG, Podkolodnaya OA, Kolchanov NA, Podkolodny NL. 2001. Inst Cytol & Genet, Lab Theoret Genet, Laurentiev Ave 10, RU-630090 Novosibirsk, Russia. Nucleosome formation potential of eukaryotic DNA: Calculation and promoters analysis. *Bioinformatics* **17:** (11) 998.

Levitsky VG, Podkolodnaya OA, Kolcharov NA, Podkolodny NL. 2001. Inst Cytol & Genet, Lab Theoret Genet, Laurentiev Ave 10, RU-630090 Novosibirsk, Russia. Nucleosome formation potential of exons, introns, and Alu repeats. *Bioinformatics* **17:** (11) 1062.

Levy S, Hannenhalli S, Workman C. 2001. Celera Genomics Corp, Informat Res, 45 West Gude Dr, Rockville, Md 20850, USA. Enrichment of regulatory signals in conserved non-coding genomic sequence. *Bioinformatics* **17:** (10) 871.

Li FG, Stormo GD. 2001. Washington Univ, Sch Med, Dept Genet, St

Copyright © 2002 John Wiley & Sons, Ltd.

Louis, Mo 63110, USA. Selection of optimal DNA oligos for gene expression arrays. *Bioinformatics* **17:** (11) 1067.
Mallios RR. 2001. Calif State Univ Fresno, Off Sponsored Projects & Res, 2615 East Clinton Ave, Fresno, Ca 93703, USA. Predicting class II MHC/peptide multi-level binding with an interative stepwise discriminant analysis meta-algorithm. *Bioinformatics* **17:** (10) 942.
Matthews LR, Vaglio P, Reboul J, Ge H, Davis BP, Garrels J, Vincent S, Vidal M*. 2001. *Harvard Univ, Sch Med, Dana Farber Canc Inst, 44 Binney St, Boston, Ma 02115, USA. Identification of potential interaction networks using sequence-based searches for conserved protein-protein interactions or "interlogs". *Genome Res* **11:** (12) 2120.
Migliavacca E, Adzhubei AA*, Peitsch MC. 2001. *GlaxoSmithKline, R&D, 10 Route Aerport, CH-1215 Geneva, Switzerland. MDB: A database system utilizing automatic construction of modules and STAR-derived universal language. *Bioinformatics* **17:** (11) 1047.
Mishra B. 2002. NYU, Courant Inst Math Sci, 251 Mercer St, New York, NY 10012, USA. Comparing genomes. *Comput Sci Eng* **4:** (1) 42.
Moller S, Schroeder M, Apweiler R. 2001. European Bioinformat Inst, Cambridge, England. Consistent integration of non-reliable heterogeneous information resources applied to the annotation of transmembrane proteins. *Comput Chem* **26:** (1) 41.
Muller PY, Studer E, Miserez AR. 2001. Univ Basel, Dept Clin Biol Sci, Res Grp Cardiovasc Genet, Vesalgasse 1, CH-4051 Basel, Switzerland. Molecular BioComputing suite: A word processor add-in for the analysis and manipulation of nucleic acid and protein sequence data. *Biotechniques* **31:** (6) 1306.
Novichkov PS, Gelfand MS*, Mironov AA. 2001. *State Sci Ctr Gosniigenet, 1 Dorozhny pr 1, RU-113545 Moscow, Russia. Gene recognition in eukaryotic DNA by comparison of genomic sequences. *Bioinformatics* **17:** (11) 1011.
Parker KC. 2002. Appl Biosyst Inc, 500 Old Connecticut Path, Framingham, Ma 01701, USA. Scoring methods in MALDI peptide mass fingerprinting: ChemScore, and the ChemApplex program. *J Am Soc Mass Spectrom* **13:** (1) 22.
Patriotis PC, Querec TD, Gruver BN, Brown TR, Patriotis C*. 2001. *Fox Chase Canc Ctr, Div Med Sci, 7701 Burholme Ave, Philadelphia, Pa 19111, USA. ArrayExplorer©, a program in visual basic for robust and accurate filter cDNA array analysis. *Biotechniques* **31:** (4) 862.
Peri S, Steen H, Pandey A*. 2001. *Univ Sthn Denmark, Ctr Expt Bioinformat, Prot Interact Lab, Odense M, Denmark. GPMAW: A software tool for analyzing proteins and peptides. *Trends Biochem Sci* **26:** (11) 687.
Phan VT, Skiena SS*. 2001. *SUNY Stony Brook, Dept Comp Sci, Stony Brook, NY 11794, USA. Dealing with errors in interactive sequencing by hybridization. *Bioinformatics* **17:** (10) 862.
Pickering SJ, Bulpitt AJ, Efford N, Gold ND, Westhead DR*. 2001. *Univ Leeds, Sch Biochem & Mol Biol, Leeds LS2 9JT, England. AI-based algorithms for protein surface comparisons. *Comput Chem* **26:** (1) 79.
Prabakaran P, An JH, Gromiha MM, Selvaraj S, Uedaira H, Kono H, Sarai A*. 2001. *RIKEN Tsukuba Inst, 3-1-1 Koyadai, Tsukuba, Ibaraki 305 0074, Japan. Thermodynamics database for protein-nucleic acid interactions (ProNIT). *Bioinformatics* **17:** (11) 1027.
Ramensky VE, Makeev VJ, Roytberg MA, Tumanyan VG. 2001. VA Engelhardt Mol Biol Inst, Vavilova 32, RU-117984 Pushchino, Moscow Region, Russia. Segmentation of long genomic sequences into domains with homogeneous composition with *BASIO* software. *Bioinformatics* **17:** (11) 1065.
Randic M, Zupan J, Novic M. 2001. 3225 Kingman Rd, Des Moines, Ia 50311, USA. On 3-D graphical representation of proteomics maps and their numerical characterization. *J Chem Inform Comput Sci* **41:** (5) 1339.
Reese MG. 2001. ValiGen, Tour Neptune, FR-92086 Paris, France. Application of a time-delay neural network to promoter annotation in the *Drosophila melanogaster* genome. *Comput Chem* **26:** (1) 51.
Riggins GJ. 2001. Duke Univ, Med Ctr, Durham, NC 27710, USA. Using Serial Analysis of gene expression to identify tumor markers and antigens. *Dis Marker* **17:** (2) 41.
Rocke DM, Durbin DB. 2001. Univ Calif Davis, Dept Appl Sci, 3011 Engn Unit 3, Davis, Ca 95616, USA. A model for measurement error for gene expression arrays. *J Comput Biol* **8:** (6) 557.
Rogozin IB, Kochetov AV, Kondrashov FA, Koonin EVC, Milanesi L. 2001. NIH/Natl Lib Med, Natl Ctr Biotechnol Informat, 8600 Rockville Pike, Bldg 38A, Rm 805, Bethesda, Md 20894, USA. Presence of ATG triplets in 5′ untranslated regions of eukaryotic cDNAs correlates with 'weak' context of the start colon. *Bioinformatics* **17:** (10) 890.
Rzhetsky A, Gomez SM. 2001. Columbia Univ, Columbia Genome Ctr, New York, NY 10032, USA. Birth of scale-free molecular networks and the number of distinct DNA and protein domains per genome. *Bioinformatics* **17:** (10) 988.
Schacherer F, Choi C, Gotze U, Krull M, Pistor S, Wingender E. 2001. German Res Ctr Biotechnol, Halchtersche Str 33, DE-38304 Wolfenbuttel, Germany. The TRANSPATH signal transduction database: A knowledge base on signal transduction networks. *Bioinformatics* **17:** (11) 1053.
Sidhu KS, Sangvanich P, Brancia FL, Sullivan AG, Gaskell SJ, Wolkenhauer O, Oliver SG*, Hubbard SJ. 2001. *Univ Manchester, Sch Biol Sci, 2-205 Stopford Bldg, Oxford Rd, Manchester M13 9PT, England. Bioinformatic assessment of mass spectrometric chemical derivatisation techniques for proteome database searching. *Proteomics* **1:** (11) 1368.
Swain MT, Kemp GJL. 2001. University Aberdeen, Department Comp Sci, Aberdeen AB24 3UE, Scotland. Modelling protein side-chain conformations using constraint logic programming. *Comput Chem* **26:** (1) 85.
Theilhaber J, Bushnell S, Jackson A, Fuchs R. 2001. Aventis Pharmaceut, Cambridge Genomics Ctr, 26 Landsdowne St, Cambridge, Ma 02139, USA. Bayesian estimation of fold-changes in the analysis of gene expression: The PFOLD algorithm. *J Comput Biol* **8:** (6) 585.
Tsai J, Voss N, Gerstein M*. 2001. *Yale Univ, Bass Ctr, Dept Biochem & Mol Biophys, 266 Whitney Ave, POB 208114, New Haven, Ct 06520, USA. Determining the minimum number of types necessary to represent the sizes of protein atoms. *Bioinformatics* **17:** (10) 949.
Turcotte M, Muggleton SH, Sternberg MJE*. 2001. *Imperial Canc Res Fund, Biomolec Modelling Lab, POB 123, 44 Lincolns Inn Fields, London WC2A 3PX, England. Generating protein three-dimensional fold signatures using inductive logic programming. *Comput Chem* **26:** (1) 57.
Wang LQ, Wu Q, Qui P, Mirza A, McGuirk M, Kirschmeier P, Greene JR, Wang YL, Pickett CB, Liu SX*. 2001. *Schering Plough Corp, Dept Tumor Biol, Res Inst, Kenilworth, NJ 07033, USA. Analyses of p53 target genes in the human genome by bioinformatic and microarray approaches. *J Biol Chem* **276:** (47) 43604.
Weinel C, Tummler B, Hilbert H, Nelson KE, Kiewitz C. 2001. Hannover Med Sch, Klin Forschergrp, OE 6711, Carl Neuberg Str 1, DE-30625 Hannover, Germany. General method of rapid Smith/Birnstiel mapping adds for gap closure in shotgun microbial genome sequencing projects: Application to *Pseudomonas putida* KT2440 (Online paper). *Nucleic Acids Res* **29:** (22) E110.
Weinstein JN, Scherf U, Lee JK, Nishizuka S, Gwadry F, Bussey AK, Kim S, Smith LH, Tanabe L, Richman S, Alexander J, Kouros-Mehr H, Maunakea A, Reinhold WC. 2002. NIH, Bldg 37, Rm 4F-28, 9000 Rockville Pike, Bethesda, Md 20892, USA. The bioinformatics of microarray gene expression profiling. *Cytometry* **47:** (1) 46.
Wheelan SJ, Church DM, Ostell JM. 2001. NIH, Natl Lib Med, Natl Ctr Biotechnol Information, Bldg 10, Bethesda, Md 20894, USA. Spidey: A tool for mRNA-to-genomic alignments. *Genome Res* **11:** (11) 1952.
Wolfinger RD, Gibson G, Wolfinger ED, Bennett L, Hamadeh H, Bushel P, Afshari C, Paules RS. 2001. SAS Inst Inc, Cary, NC 27513, USA. Assessing gene significance from cDNA microarray expression data *via* mixed models. *J Comput Biol* **8:** (6) 625.
Wu W, Wildsmith SE, Winkley AJ, Yallop R, Elcock FJ, Bugelski PJ. 2001. GlaxoSmithKline Safety Assessment, The Frythe, Welwyn Garden City AL6 9AR, England. Chemometric strategies for normalisation of gene expression data obtained from cDNA microarrays. *Anal Chim Acta* **446:** (1-2) 451.
Yeung KY, Fraley C, Murua A, Raftery AE, Ruzzo WL. 2001. Univ Washington, Box 352350, Seattle, Wa 98195, USA. Model-based clustering and data transformations for gene expression data. *Bioinformatics* **17:** (10) 977.
Yuan J, Liu Y, Wang YH, Xie GC, Blevins R. 2001. Merck & Co Inc, Dept Bioinformat, POB 2000, Rahway, NJ 07065, USA. Genome analysis with gene-indexing databases. *Pharmacol Ther* **91:** (2) 115.
Zhang PM, Li MMZ, Elledge SJ*. 2002. *Baylor Coll Med, Dept Physiol & Mol Biophys, Houston, Tx 77030, USA. Towards genetic genome projects: Genomic library screening and gene-targeting vector construction in a single step. *Nat Genet* **30:** (1) 31.

Copyright © 2002 John Wiley & Sons, Ltd. 
